# Supplementary material for: Optimising a couples-focused intervention to increase couples’ HIV testing and counselling using the person-based approach: a qualitative study in Kwa-Zulu Natal, South Africa
Source: BMJ Open. 2021 Dec 1;11(12):e047408. doi: 10.1136/bmjopen-2020-047408 (PMC8638156; doi:10.1136/bmjopen-2020-047408)
Supplement: Supplementary data [file bmjopen-2020-047408supp002.pdf]

Supplementary File 2: Semi-structured interview schedule for participants in the couples-focused intervention

**A. Introduction and thanks**
My name is ************ and I am working for the 'Egugu Lethu project' which is being conducted by the Human Sciences Research Council and the University of Southampton, UK.<br><br>First, let me thank you for agreeing to speak to me today.
**B. Project explanations**
The overall aim of the 'Egugu Lethu project is to improve the couples counselling intervention that we used in the Uthando Lwethu study and to conduct a new study to test the improved intervention success in promoting couples HIV testing and counselling.<br><br>This interview is part of the first steps towards improving the previously tested intervention. We are conducting interviews with individual members of couples from the Uthando Lwethu study to explore their experiences of the intervention and it's role, if any, in contributing to their decision regarding uptake of couples HIV testing and counselling.

Interview explanations

Today I would like to ask you about your experiences of participating in the Uthando Lwethu Study. We would really like to hear your experiences, opinions and observations. I will ask you a series of questions from my guideline, but please feel free to add in extra comments that you think are important.
[I will be taping / I will not be taping] this interview and taking a few notes to aid my memory as we go along but I will not write down your name or the name of your partner.
I will not be using a questionnaire but have a list of topics that I'd like us to try and cover today. Think of our session as a conversation and that you can ask me questions too.

Great, so now we have completed the formalities, is it fine with you that we start?

What is your name?

And the name of your partner who participated with you in Uthando Lwethu Study?

General experience of the Uthando Lwethu Study

Before we start talking in detail about your experiences of specific parts of the Uthando Lwethu Study, can you tell me about the first day when you were approached about the study? What was that like for you?
Can you remember why you decided to sign up for the study?

Morton K, *et al. BMJ Open* 2021; 11:e047408. doi: 10.1136/bmjopen-2020-047408

I’m really interested in hearing about your experiences of the Uthando Lwethu study, Can you tell me what you remember about being in the study?

How did you find the sessions?

Does anything stand out in your memory about the study? What do you remember and why?

Can you tell me about what difference Uthando Lwethu made for you, if any?

Experience of the first group session

As a quick reminder, the first group session was where we gave some information about health to the group of couples attending together and at the end we had the lottery where you learned you and your partner were going to receive the Uthando Lwethu couples counselling sessions .

Can you tell me all about what the first group session was like for you?

[Note to interviewer: information given was on TB, reproductive health/HIV), and a list of community resources for health care and treatment was given]

What information did you find most helpful? And why?

What other information would you have liked?

How was it to see other couples from your area at the same session? Did you know anyone else there? Who?

Can you remember how you sat in the first session? Did you sit with your partner, or with other men/women?

How did you interact with other couples from your area?

Can you tell us about anything you remember learning in the first session?

Experience of the second group session

The second group session was where you met in [men-only / female only] groups and discussed roles, violence and power within relationships. There was also skills training for communication and risk reduction. What was your experience of that second group session?

What was most helpful in this session? Why?

What other information would you have liked? Why?

Can you tell me about any conversations you had with your partner about that session afterwards?

What did you discuss? Once off or repeatedly?

Were there any particular topics that you discussed in particular? [prompt for specific exercises / content that was included in the session to help participants remember]

Was one same gender group session enough or would you have liked more? Why?

Couples sessions

The next section is dependent on whether they attended the couples' sessions. Only one block of questions from this section is applicable in any particular individual interview.

a. For those who did NOT attend couples sessions at all

If you remember, you were then offered 4 couples' sessions *with* <counsellor's name> Can you tell me about why you did not attend the couples' sessions that were on offer?

How did you reach that decision?

Was this your choice or your partner's, or mutual?

What were your main concerns about the couples' sessions?

Could the study have done something differently that might have changed your mind? Or your partner's mind? Or made it possible logistically?

b. For those who attended one or more couples sessions

What was your main reason for deciding to attend the couples' sessions?

How did you reach that decision?

Did you have any concerns or worries about attending?

Do you remember your counsellor? [check they mean the same person recorded on page 1]. I am interested in how you felt about the couples' sessions that you attended.

If I asked you to describe ~~them~~ *the sessions in one or two sentences*, what would you say?

Morton K, *et al. BMJ Open* 2021; 11:e047408. doi: 10.1136/bmjopen-2020-047408

*Did you feel you were able to communicate your point of view and thoughts to your **partner** in the counselling session? And did your partner? Can you tell me about why?*

*Did you feel you were able to communicate your point of view and thoughts to the **counselor** in the counselling session? Can you tell me about why?*

How do you think your partner felt about it?

Was there anything you would change?

***[If they used all 4 sessions]:** Was the number of sessions enough*

*[**If they used less than 4 sessions]:** Can you tell me about why you stopped attending when you did?*

Would having an additional session made a difference? Was another session likely to have changed your decision about testing?

Can you tell us about anything you remember learning from the sessions? [prompt for specific tasks or exercises / content to help participants remember]

Did you discuss anything about the session or from a session with your partner afterwards? What did you talk about?

Do you remember some of the homework exercises that the counsellor gave you? How did you and your partner get on with the homework?

(If still together) Can you tell me how you feel about communicating with your partner now?

Are you still practicing the skills taught? Why or why not? If not, how long after did you stop using the skills?

Cohabitation

The next section is dependent on the couple cohabitation status at the time of their participation in the Uthando Lwethu study. Only one block of questions from this section is applicable in any particular individual interview.

a. For those cohabiting at the time

At the time of the Uthando Lwethu study, you were living with your partner.

Can you tell me about what it was like doing the Uthando Lwethu study while you were living at home with your partner?

Did you want to talk to your partner about Uthando Lwethu?

If yes – did you have the opportunity to talk to them at home?

If no – why not?

Was anything different at home when you were both doing Uthando Lwethu?

b. For those NOT cohabiting at the time

At the time of the Uthando Lwethu study, you were not living with your partner.

Can you tell me about what it was like doing the Uthando Lwethu study while you were living separately from your partner?

Did you want to talk to your partner about Uthando Lwethu?

If yes – did you have the opportunity to talk to them? Where did the conversations happen?

If no – why not?

Was anything different when you met up while you were both doing Uthando Lwethu?

HIV testing

The next section is for ALL individuals

What do you think about HIV testing?

And testing with a partner together?

What are your main concerns about HIV testing?

How could we address those concerns?

The next section is dependent on whether the couple tested for HIV as a couple during the Uthando Lwethu study. Only one block of questions from this section is applicable in any particular individual interview.

a. For those who did not test for HIV as a couple during the Uthando Lwethu Study

I'm really interested in your decision around whether to test for HIV with your partner. It looks like you decided not to test together during the study, can you tell me more about that decision?

Did you as a couple discuss testing? Why or why not? How did you reach a decision?
How did you feel about getting tested?

How did your partner feel?

Morton K, *et al. BMJ Open* 2021; 11:e047408. doi: 10.1136/bmjopen-2020-047408

What were your main concerns? What could be done to address those concerns? Eg more information? sessions? support?

What do you think might have been the benefits of testing for you?

How did you resolve any conflict with your partner?

Did you test together anytime after the study had finished? How long after the study?

Do you know your partner's status? How do you know this?

Do you think the group sessions or couples counseling sessions contributed to your / your partner's decision?

b. For those who tested for HIV as a couple during the Uthando Lwethu Study

Have you ever tested together before Uthando Lwethu?
If yes - how did you decide to do this?

Can you tell me about why you decided to test together during the Uthando Lwethu study?

How did you reach a decision?

How did you feel about getting tested?

How did your partner feel?
Did you have any concerns about the testing? What could be done to address those concerns? Eg more information? sessions? support?

How did you resolve any conflict with your partner about deciding whether to get tested?

How did you arrange the testing?

How did you feel after the testing?

Did you talk about it with your partner? What do you think were the benefits of testing for you?

How would you encourage other couples to have HIV testing?

Do you think the group sessions or couples counseling sessions contributed to your / your partner's decision?

If yes: What part of the intervention encouraged you to be tested?

If not, why not?

For all individuals

In the new study, we may offer testing for sexually transmitted infections (STIs), specifically testing for chlamydia, syphilis, gonorrhoea and trichomoniasis. However we don't know if this would be useful for people, or what some of the concerns might be in having STI testing.

Can you tell me what you think about the idea of testing couples for sexually transmitted infections?

If we had also invited you to consider STI testing as part of the couples HIV testing session, how would you have felt about that?

Would you have taken up an offer for STI testing? Why/why not?

The STI testing would mean giving a sample that would be sent away to the labs for testing and you and your partner would come back to receive the results, For men, this would involve providing a urine sample. How would you feel about that?

For women, this would mean privately taking a vaginal swab. Many women do this themselves, or the nurse could help you with if you wanted. How would you feel about that?

Overall, what do you think about the Uthando Lwethu study for other people?

We are hoping to make some changes to Uthando Lwethu to make it even better for people. Can you think of any other health or relationship topics you would have liked to know more about in group session 1, group session 2, or the counselling sessions?

Can you think of anything that would have made Uthando Lwethu better for you?

And was there any part of Uthando Lwethu that you think was not needed or was not helpful to you?

Morton K, *et al. BMJ Open* 2021; 11:e047408. doi: 10.1136/bmjopen-2020-047408
