# Supplementary material for: Optimising a couples-focused intervention to increase couples’ HIV testing and counselling using the person-based approach: a qualitative study in Kwa-Zulu Natal, South Africa
Source: BMJ Open. 2021 Dec 1;11(12):e047408. doi: 10.1136/bmjopen-2020-047408 (PMC8638156; doi:10.1136/bmjopen-2020-047408)
Supplement: Supplementary data [file bmjopen-2020-047408supp003.pdf]

Supplementary File 3. Semi-structured interview schedule for staff members.

A. Introduction and thanks
My name is ************ and I am working for the 'Egugu Lethu project' which is being conducted by the Human Sciences Research Council and the University of Southampton, UK.<br><br>First, let me thank you for agreeing to speak to me today.
**B. Project explanations**
The overall aim of the Egugu Lethu project is to improve the couples counselling intervention that we used in the Uthando Lwethu study and to conduct a new study to test the improved intervention success in promoting couples HIV testing and counselling.<br><br>The first step of optimising the previously tested intervention is to conduct interviews with individual members of couples from the Uthando Lwethu study and with staff members who were involved in delivering the intervention components. In staff interviews we will discuss staff experiences of the previous study and possible barriers to couples' decisions to test for HIV together.

Interview explanations

Today I would like to ask you about your experiences as an Uthando Lwethu Study staff member. We would really like to hear your experiences, opinions and observations. I will ask you a series of questions from my guideline, but please feel free to add in extra comments that you think are important. [I will be taping / I will not be taping] this interview and taking a few notes to aid my memory as we go along but I will not write down your name. I will not be using a questionnaire but have a list of topics that I'd like us to try and cover today. Think of our session as a conversation and that you can ask me questions too.

Great, so now we have completed the formalities, is it fine with you that we start?

What is your name?

What was your role in the Uthando Lwethu Study: Couples counsellor or part of the group sessions or counsellor for the HIV testing and counselling?

Can you tell me a bit about your background? I'd like to understand what your job is outside of Uthando Lwethu?
Can you tell me about the training you've had previously?
What was different in Uthando Lwethu from your usual counselling/facilitating role?
What was the same?

General experience of the Uthando Lwethu Study

Morton K, et al. BMJ Open 2021; 11:e047408. doi: 10.1136/bmjopen-2020-047408

Before we start talking in detail about your experiences and perceptions of specific parts of the Uthando Lwethu Study, I would like to hear what was it like being part of the study team overall?

How did you find the training you received before becoming a facilitator/counsellor for Uthando Lwethu?

The next section is dependent on which role the staff member had. Only one block of questions from this section is applicable in any particular individual interview.

For staff who attended group sessions

How many first group sessions did you attend? What was your role in the first group sessions?

what was your role in the second group sessions?

We're interested in how to improve the intervention for couples, so the next few questions are to try and find out more about how it worked last time, and what we could do better going forwards.

How did you find the first group sessions?

How did you find the second group sessions?

Did you notice any differences between the two sessions?

Can you recall what happened in a typical second group session?

What was it like following the manual for Uthando Lwethu during the second group session?

Were there any activities that stood out for you as being particularly important in the second group session?

Were there any activities that you remember being challenging for some people in the second group session?

How engaged did you feel people were? Can you tell me a story about any memorable moments you recall?

Are there any particular benefits/negatives to the single gender session?

How did the single gender sessions vary between groups?

What was it like to deliver this information to females/males as a man/woman yourself? Did the same / different gender dynamic work well or not really?

Could session 2 be reinforced by homework If we introduced homework at this session? do you think it would work best done as a couple of individually?

Did you feel there were any differences in the level of participation or questions between sessions?

How important do you think these group sessions were for encouraging couples to attend HIV testing together?

Do you think there was anything about the first or second group session that didn't work very well, that you think we should change for the next study? *[prompt for organizational or staff changes vs content]*

If **not** already mentioned, ask about:

In the first group session, how do you think couples found the information on:

- TB
- HIV
- HIV testing
- Contraception
- Alcohol

In the second group session, how do you think participants found the:

- Discussion around long-term relationships
- Condom demonstration
- Discussions about gender and power imbalance
- Practising communication skills

In the new study, we may offer testing for sexually transmitted infections (STIs), specifically testing for chlamydia, syphilis, gonorrhoea and trichomoniasis. Would any changes be needed in the way Group sessions 1 and 2 are conducted or their content if STI testing were offered alongside HIV testing and counselling?

How do you think couples would feel about testing for STIs together?
For men, it would involve providing a urine sample, and for women it would mean taking a vaginal swab. The samples are sent off to a lab, so the couple would need to come back to receive their results. How do you think this would work for couples in Uthando Lwethu?

For staff who conducted couples counselling sessions

Approximately, how many couples did you counsel?

How did you feel about the couples' counselling sessions that you led?

How do you think they were experienced by the participating couples?

Within those counselling sessions, how did you find it interacting with the female partner, as a female/male counsellor? And what about working with the male partner? Why?

Did the interaction with different couples vary? In what way did it vary and why?

Can you tell me about any reasons you can think of why some couples got more out of the counselling than others?

How do you think couples found the number of counselling sessions? Do you think having an additional session would make a difference / likely to have changed couples' decisions about testing?

How do you think the couples felt about practicing the speaker-listener technique between sessions?

Which exercises in the counselling sessions were particularly helpful for couples?

Were there any activities in the counselling sessions that you remember being challenging for some couples?

What was it like following the intervention manual in the couples counselling session?

If **not** already mentioned, ask the following:
In the couples counselling sessions, what do you think it was like for couples:

- Identifying and talking about their expectations and barriers around HIV testing
- Practising the speaker listener technique with their partner
- Planning solutions to problems together
- Setting some personal goals
- Doing the 'forgetting something good' activity, where they noticed what helpful things their partner has done for them recently
- Doing the 'words of the heart' activity where couples chose words that describe their relationship

If the participant only talks about positive examples where couples liked these things, ask:

- *Were there any couples who struggled to do that?*

- *If so, what was that like for you?*

Why do you think some couples didn't go for HIV testing? What do you think were the barriers for them?

Can you tell me about a couple who did go for testing? Why do you think they decided to get tested?

In the new study, we may offer testing for sexually transmitted infections (STIs), specifically testing for chlamydia, syphilis, gonorrhoea and trichomoniasis. Would any changes be needed in the way the couples counselling sessions are conducted or their content if STI testing were offered alongside HIV testing and counselling?

How do you think couples would feel about testing for STIs together?
For men, it would involve providing a urine sample, and for women it would mean taking a vaginal swab. The samples are sent off to a lab, so the couple would need to come back to receive their results. How do you think this would work for couples in Uthando Lwethu?

For staff who conducted couples HIV testing and counselling during the Uthando Lwethu Study

Approximately how many couples HIV testing and counselling sessions did you conduct during Uthando Lwethu Study?

How did you feel about the couples' HIV testing and counselling sessions that you conducted?

Did the couples discuss with you how they came to the decision to test for HIV together? What reason(s) did they give?

In the new study, we may offer testing for sexually transmitted infections (STIs), specifically testing for chlamydia, syphilis, gonorrhoea and trichomoniasisdo you think offering STI testing as part of the session would change couples' interest in testing for HIV together?

To what extent do you think including STI testing in the couples testing session would change this session for you as counsellor?

How do you think couples would feel about testing for STIs together?
For men, it would involve providing a urine sample, and for women it would mean taking a vaginal swab. The samples are sent off to a lab, so the couple would need to come back to receive their results. How do you think this would work for couples in Uthando Lwethu?

For all staff at the end:

Did you have any contact with couples after ***their*** *participation in the study had ended?*

What was the nature of those interactions? [Probe for friendly / casual, neighbour, asking advice, sought out staff member's skill as counsellor etc.]

Morton K, *et al. BMJ Open* 2021; 11:e047408. doi: 10.1136/bmjopen-2020-047408
