# Supplementary material for: Optimising a couples-focused intervention to increase couples’ HIV testing and counselling using the person-based approach: a qualitative study in Kwa-Zulu Natal, South Africa
Source: BMJ Open. 2021 Dec 1;11(12):e047408. doi: 10.1136/bmjopen-2020-047408 (PMC8638156; doi:10.1136/bmjopen-2020-047408)
Supplement: Author's manuscript [file bmjopen-2020-047408.draft_revisions.pdf]

BMJ Open is committed to open peer review. As part of this commitment we make the peer review history of every article we publish publicly available.

When an article is published we post the peer reviewers' comments and the authors' responses online. We also post the versions of the paper that were used during peer review. These are the versions that the peer review comments apply to.

The versions of the paper that follow are the versions that were submitted during the peer review process. They are not the versions of record or the final published versions. They should not be cited or distributed as the published version of this manuscript.

BMJ Open is an open access journal and the full, final, typeset and author-corrected version of record of the manuscript is available on our site with no access controls, subscription charges or pay-per-view fees (http://bmjopen.bmj.com).

If you have any questions on BMJ Open's open peer review process please email info.bmjopen@bmj.com

BMJ Open

Optimising a couples-focused intervention to increase couples' HIV testing and counselling using the person-based approach: A qualitative study in Kwa-Zulu Natal, South Africa.

Journal:	*BMJ Open*
Manuscript ID	bmjopen-2020-047408
Article Type:	Original research
Date Submitted by the Author:	27-Nov-2020

Complete List of Authors:	Morton, Katherine; University of Southampton, Psychology<br>Mhlakwaphalwa, Tembeka; Human Sciences Research Council<br>Msimango, Lindani; Human Sciences Research Council<br>van Heerden , Alastair ; Human Sciences Research Council; University of the Witwatersrand Faculty of Health Sciences, School of Clinical Medicine<br>Ngubane, Thulani; Human Sciences Research Council<br>Joseph, Philip; Human Sciences Research Council<br>Ngcobo, Nathi; Human Sciences Research Council<br>Feng, Z; University of Southampton, Primary Care and Population Sciences<br>Hosegood, Victoria; University of Southampton, Department of Social Statistics and Demography, Faculty of Social Sciences<br>van Rooyen, Heidi; Human Sciences Research Council; University of the Witwatersrand<br>McGrath, Nuala; University of Southampton, School of Primary Care, Population Sciences and Medical Education, Faculty of Medicine; University of Southampton, Department of Social Statistics & Demography, Faculty of Social Sciences

Keywords:	HIV & AIDS < INFECTIOUS DISEASES, PUBLIC HEALTH, QUALITATIVE RESEARCH

SCHOLARONE™
Manuscripts

BMJ

I, the Submitting Author has the right to grant and does grant on behalf of all authors of the Work (as defined in the below author licence), an exclusive licence and/or a non-exclusive licence for contributions from authors who are: i) UK Crown employees; ii) where BMJ has agreed a CC-BY licence shall apply, and/or iii) in accordance with the terms applicable for US Federal Government officers or employees acting as part of their official duties; on a worldwide, perpetual, irrevocable, royalty-free basis to BMJ Publishing Group Ltd ("BMJ") its licensees and where the relevant Journal is co-owned by BMJ to the co-owners of the Journal, to publish the Work in this journal and any other BMJ products and to exploit all rights, as set out in our licence.

The Submitting Author accepts and understands that any supply made under these terms is made by BMJ to the Submitting Author unless you are acting as an employee on behalf of your employer or a postgraduate student of an affiliated institution which is paying any applicable article publishing charge ("APC") for Open Access articles. Where the Submitting Author wishes to make the Work available on an Open Access basis (and intends to pay the relevant APC), the terms of reuse of such Open Access shall be governed by a Creative Commons licence – details of these licences and which Creative Commons licence will apply to this Work are set out in our licence referred to above.

Other than as permitted in any relevant BMJ Author's Self Archiving Policies, I confirm this Work has not been accepted for publication elsewhere, is not being considered for publication elsewhere and does not duplicate material already published. I confirm all authors consent to publication of this Work and authorise the granting of this licence.

For peer review only

Optimising a couples-focused intervention to increase couples' HIV testing and counselling using the person-based approach: A qualitative study in Kwa-Zulu Natal, South Africa.

Kate Morton[1] k.s.morton@soton.ac.uk

Tembeka Mhlakwaphalwa[2] tembekam23@gmail.com

Lindani Msimango[2] LIMsimango@hsrc.ac.za

Alastair Van Heerden[2, 3] avanheerden@hsrc.ac.za

Thulani Ngubane[2] tngubane@hsrc.ac.za

Phillip Joseph[2] pjoseph@hsrc.ac.za

Nkosinathi Ngcobo[2] NNgcobo@hsrc.ac.za

Zhixin Feng[4] Z.Feng@soton.ac.uk

Victoria Hosegood[5] V.Hosegood@soton.ac.uk

Heidi Van Rooyen[2,3] hvanrooyen@hsrc.ac.za

Nuala McGrath[4,5] N.McGrath@soton.ac.uk

1. School of Psychology, University of Southampton, UK.

2. Human Sciences Research Council, Sweetwaters, KwaZulu-Natal, South Africa.

3. SAMRC/WITS Developmental Pathways for Health Research Unit Department of Paediatrics, School of Clinical Medicine, Faculty of Health Sciences, University of the Witwatersrand, Johannesburg, South Africa.

For peer review only - http://bmjopen.bmj.com/site/about/guidelines.xhtml

4. School of Primary Care, Population Sciences and Medical Education, Faculty of Medicine, University of Southampton, UK

5. Department of Social Statistics & Demography, Faculty of Social Sciences, University of Southampton, UK

For peer review only

For peer review only - http://bmjopen.bmj.com/site/about/guidelines.xhtml

Abstract:

Couples HIV testing and counselling (CHTC) facilitates mutual disclosure in a safe and supported environment. A couples-focused intervention comprised of two group sessions and four couples counselling sessions was previously shown to significantly increase uptake to CHTC in Kwa-Zulu Natal, South Africa. However, more than half of couples participating in the intervention still chose not to test together during follow-up. This qualitative study explored barriers to CHTC amongst intervention participants, and possible optimisations to the intervention to increase uptake to couples' HIV testing in this setting.

Qualitative interviews were conducted with 20 couples who had participated in the intervention and five staff members delivering the intervention. Partners were interviewed individually by researchers of the same gender. The transcripts were analysed using the table of changes from the person-based approach. Proposed optimisations were discussed with a community group to ensure the intervention was as persuasive and acceptable as possible.

Many couples had concerns about the impact of CHTC on their relationship due to an implied lack of trust, which was also recognised as a barrier by staff members. Single-gender group discussions facilitated by peer mentors, alongside open discussion of personal barriers to CHTC during couples' counselling sessions could help overcome this barrier. Additional training for staff in open questioning techniques could help them feel more comfortable to explore couples' perceived barriers to CHTC, rather than advising couples to test. A logic model was developed to show anticipated mechanisms through which the optimised intervention would increase uptake to CHTC, including increasing knowledge, increasing positive outcome beliefs, and managing negative emotions.

In-depth qualitative research informed optimisations to a couples-focused intervention for further evaluation in South Africa to encourage uptake to CHTC. Suggestions are made for optimal methods

For peer review only - http://bmjopen.bmj.com/site/about/guidelines.xhtml

to gain open feedback on intervention experiences where participants may be reluctant to share negative views.

Key words: Couples-focused, intervention, Couples HIV Counselling and Testing, person-based approach

For peer review only

For peer review only - http://bmjopen.bmj.com/site/about/guidelines.xhtml

Strengths and Limitations of this study

- Conducting qualitative interviews with partners separately, in their own homes, and with a researcher of the same gender helped promote rapport and facilitated open reflections about their relationship.
- Interviewing staff members responsible for delivering the intervention in addition to couples participating in the intervention provided a more in-depth understanding of potential barriers to CHTC.
- The Table of Changes from the Person-Based Approach provided a rigorous method to analyse the qualitative interviews, while the use of guiding principles and a logic model helped ensure that optimisations were grounded in the specific psychosocial context of this population, and drew on theoretical constructs.
- Conducting the interviews five years after the intervention had been delivered may have limited participants' ability to accurately recall their experiences and perceptions.
- Many participants appeared reluctant to share any negative views of the intervention, and alternative methods such as the use of vignettes may have helped obtain more critical feedback about the couples-focused intervention.

For peer review only

1. Introduction

Couples HIV Testing and Counselling (CHTC) is recommended by the World Health Organisation (1), however, very few couples in South Africa have tested together (2, 3). This is against a background of continued high levels of HIV prevalence, particularly in KwaZulu-Natal (KZN), South Africa. In a large community-based survey of adults in KZN, 19% of men and 41% of women were living with HIV in 2017 (4). The majority of couples in stable sexual relationships reported being unaware of their partner's HIV status (57%) (5). CHTC can contribute to reductions in HIV incidence as a compliment to the universal test and treat policy in South Africa (6) and individual focused testing campaigns (7).

In 2012-2015, a couples-focused intervention (*'Uthando Lwethu')* was shown to significantly increase uptake to CHTC, with 42% of couples attending CHTC within a 9-month follow-up period compared with 12% in the control group (8). Nonetheless, over half of couples receiving the intervention chose not to test within the study period. In order to optimise the couples-focused intervention for future implementation, we undertook further supplementary research to explore how the intervention content and delivery could be modified to support couples to feel ready for CHTC. This paper presents the findings from a qualitative study with couples and staff involved in the Randomised Controlled Trial (RCT) of the Uthando Lwethu intervention. The person-based approach (PBA) (Yardley et al. 2015) was adopted, which complements evidence and theory in developing and optimising effective interventions and has been used to successfully create behaviour change interventions which are feasible, persuasive, motivating and engaging (9). This approach recommends conducting in-depth research to develop an understanding of the target population's psychosocial context and underlying beliefs which could influence engagement with behaviour change. Guiding principles are developed to describe specific behavioural barriers for a given population and context and identify particular intervention features to overcome these barriers. In addition, this intervention optimisation process was informed by recent evidence regarding

For peer review only - http://bmjopen.bmj.com/site/about/guidelines.xhtml

facilitators to CHTC (10-12), and a consideration of the theoretical mechanisms through which the optimised intervention might impact on couples' readiness to undertake CHTC.

Our research questions were as follows:

a. What barriers do couples and staff perceive to engaging in CHTC during a couples-focused intervention in KwaZulu-Natal?
b. How can these barriers be addressed to optimise the intervention to support CHTC uptake?
c. What are the theorised mechanisms of action through which the optimised intervention is anticipated to change behaviour?

2. Methods

2.1 Optimisation study design

This was a qualitative study using semi-structured interviews to explore our first two research questions, informing the development of guiding principles to define how the intervention would be optimised to address specific behavioural barriers in this population and context. A logic model was then developed to address our third research question and describe how the optimised intervention was theorised to promote CHTC uptake. We report the optimisation process using the GUIDED checklist (Guidance for reporting intervention development studies in health research) in line with best practice for reporting intervention development (13). The COREQ checklist was used for reporting (Supplementary File 1). Approval was granted by the Human Sciences Research Council (REC 2/19/10/11b) and the University of Southampton (reference number: ERGO 48702) ethics committees.

2.2 Uthando Lwethu Intervention, prior to optimisation

For peer review only - http://bmjopen.bmj.com/site/about/guidelines.xhtml

As this study aimed to describe the optimisation of a couples-focused intervention to promote CHTC, the original content and delivery of the Uthando Lwethu intervention trialled in the 2012-2015 randomised controlled trial (14) is described here.

Uthando Lwethu was a couples-focused intervention comprised of two group sessions and four couples counselling sessions which aimed to increase CHTC uptake. It was developed based on interdependence theory which states that positive relationship dynamics can lead to a transformation of motivation, in which couples are motivated to engage in health behaviours due to a focus on the relationship rather than an individual perspective. It was theorised that couples will be more willing to engage in CHTC if they have greater commitment and trust in their relationship (14). The intervention was adapted from the Prevention and Relationship Enhancement Programme (PREP) which aims to improve relationship dynamics through education in problem-solving and communication skills (15).

The intervention was delivered in a community setting in KZN to couples who had been in a relationship for at least six months. Marriage rates are low in this region and many couples in established partnerships are not cohabiting (16).

Table 1 provides a summary of the content of each session. The full intervention development process is described elsewhere (14).

<insert Table 1 here>

2.3 Optimisation study participants

A sub-sample of the 168 couples who took part in the Uthando Lwethu intervention were invited to qualitative interviews (n=20 couples). Couples were purposively sampled according to their engagement; defined in terms of how many counselling sessions the couple attended, and whether

For peer review only - http://bmjopen.bmj.com/site/about/guidelines.xhtml

or not they engaged in the primary outcome of CHTC. This enabled us to explore diverse perceptions of the intervention, and explore why couples chose to test or not to test.

In addition, five staff members who delivered the intervention were interviewed.

2.4 Recruitment procedures

Recruitment was conducted indirectly by community members who had acted as community advisory board members during the Uthando Lwethu trial. When couples were still in the local area, the community member gave each partner a card inviting them to contact the research team if they were interested in discussing their experiences of the intervention. Thirty two of the 36 couples who were invited to the study agreed to participate, of whom 20 couples were interviewed.

The staff members were invited to interview opportunistically based on availability.

2.5 Interview procedures

Semi-structured qualitative interviews were conducted face-to-face in the community by a gender-matched qualitative researcher (TM; female, MSc or LM; male, MSc). TM and LM were employed as research assistants at the time, and were trained in qualitative interviewing skills by KM. Partners were interviewed separately, and where possible, simultaneously. The interviews took place from August-October 2019, and informed consent was taken at the start of each interview.

Participants' experiences of the two group sessions were explored at the start of the interview. Questions were then tailored according to whether the couple had attended counselling and/or CHTC, either to explore their experiences of these events or to ask how they decided not to attend (Supplementary File 2). Participants were reimbursed 120 ZAR ($8). Interviews were audio-recorded and researchers made field notes after each interview which were discussed with the team.

The interviews were conducted in isiZulu and translated into English during transcription either by the researcher or a research assistant. The researchers read the English translation of each interview

For peer review only - http://bmjopen.bmj.com/site/about/guidelines.xhtml

they had conducted to ensure they were happy this was an accurate record. Researchers completed a debrief form after each interview to encourage reflection.

The staff interviews were conducted in English and transcribed verbatim. One interview was conducted in person (LM) while the other four were conducted from the UK by video call (KM; female, PhD, employed as a health psychology researcher). Informed consent was taken in advance on site. The staff interview schedule explored experiences of delivering the intervention (Supplementary File 3).

2.6 Patient and Public Involvement

A community working group of eight volunteers from the local community contributed to the interpretation of interview findings and decisions about optimisations to the intervention, during an in-depth half-day workshop. The group included one couple who had taken part in the Uthando Lwethu intervention, one couple who were part of the community working group during the trial, and four individuals who were new to the intervention.

In addition, a participant feedback event was organised in which findings from the qualitative interviews were presented back to couples and staff members who had participated in interviews, and a discussion held about their perceptions of the findings.

2.7 Analysis

The interviews were analysed by KM using the table of changes from the PBA (Bradbury 2018). The table of changes provides a technique for rapid qualitative analysis involving the collation of positive and negative quotes about each aspect of the intervention, in order to identify underlying beliefs that could influence engagement with the intervention and target behaviours (i.e. CHTC).

Guiding principles were developed based on the detailed understanding of barriers to CHTC built up during the qualitative interviews, along with the team's expertise in evidence and theory relating to

For peer review only - http://bmjopen.bmj.com/site/about/guidelines.xhtml

CHTC. This was an iterative process which aimed to identify key objectives for the intervention to achieve in this context, alongside key features that could be used to achieve these objectives. Possible optimisations were proposed to overcome barriers, informed by the guiding principles. The optimisations were discussed in detail with the study team and community working group.

A logic model was developed to show how the optimised intervention components were anticipated to influence CHTC uptake. The optimised intervention components were mapped on to constructs from the Theoretical Domains Framework (TDF) (Cane et al. 2012) and Capability Opportunity Motivation-Behaviour (COM-B) model (Michie et al. 2011). The TDF was chosen as this identified 14 domains from across a wide range of behaviour change theories, and uses common terminology to describe approaches to behaviour change.

For peer review only

3. Results

Forty-one participants (aged 25-59 years) took part in an individual interview (20 couples and 1 woman whose partner could not be contacted, from Group B). The mean interview duration was 57 minutes.

Four couples were interviewed from each of the following five target groups:

a. Attended CHTC after attending the first couples counselling session
b. Attended CHTC after attending 2-4 couples counselling sessions
c. Attended 4 couples counselling sessions but did not attend CHTC
d. Attended <4 couples counselling sessions and did not attend CHTC
e. Did not attend any couples counselling sessions or CHTC

The staff members interviewed included four staff members who delivered group sessions and couples counselling, and the study manager.

For peer review only - http://bmjopen.bmj.com/site/about/guidelines.xhtml

3.1 What barriers do couples and staff perceive to engaging in CHTC during a couples-focused intervention in KwaZulu-Natal?

3.1.1 Concerns about implications of CHTC for the relationship

A common barrier raised during the interviews was that discussing HIV testing in a long-term relationship implies a lack of trust, and that testing together could lead to arguments or even the breakdown of the relationship.

'*Yes, we would talk about it [testing]. He said that it means that I do not trust him, why else would I talk about this thing. You would see from the way he responds that you should not go any further with it.'*

(C0011, female aged <30 years, Group D)

These concerns about trust and blame were reflected in the staff interviews too.

'*I think there's a lot of barriers when in a couple there's STI because there's a lot of blame. There's a lot of feeling that someone is having an affair so people they don't…I think they won't be comfortable*' (Staff member 4, counsellor and facilitator)

3.1.2 Fears about receiving an HIV positive diagnosis

There was some evidence from the interviews that fear about test results was also a barrier to CHTC, suggesting it is important for couples to understand the support available to them if they had a positive result.

'*Sometimes you see other people getting sick and you tell yourself that this cannot make you sick if you do not know. What makes you sick is knowing. It is thing like that…. You end up realizing that you are fooling yourself. It is that. It is being scared of the truth*'. (C4510, Male aged 40-50 years, Group A)

For peer review only - http://bmjopen.bmj.com/site/about/guidelines.xhtml

3.1.3 Benefits of open discussion in single-gender groups

The single-gender group session was valued by men and women as a chance to talk more openly about relationship issues.

'*Do you know how difficult it is to talk when your boyfriend is here? You cannot be open to talk about whatever, but when you are with other women you can talk about whatever concerning him*' (C0011, Female aged <30 years, group D)

Staff members also noticed that participants were more willing to have open discussions during the single-gender group session.

3.1.4 Intervention facilitators providing advice to couples

The interviews with couples counsellors suggested that their investment in positive outcomes for the couple could lead them to provide advice and try to persuade couples to engage in CHTC.

'*you speak and speak and speak and encouraging them about honesty and trust and everything but still tomorrow, no, I'm not ready to do the testing..... I kept on encouraging them that most of the people are testing negative, just to make them think about it*' (Staff member 1, counsellor and facilitator)

3.1.5 In order to inform the process of optimising the intervention, guiding principles were developed based on these findings regarding the main barriers to CHTC, see Table 2. These emphasise that the intervention needs to increase intimacy between couples, encourage effective communication about CHTC, and increase understanding. Key intervention features to achieve the design objectives were identified, which helped inform the optimisations to the intervention described below in section 3.2.

For peer review only - http://bmjopen.bmj.com/site/about/guidelines.xhtml

<Insert Table 2 here>

3.2 How can these barriers be addressed to optimise the intervention to support CHTC uptake?

3.2.1 Optimisation to overcome concerns about implications of CHTC for the relationship

In order to increase knowledge and understanding of the benefits of CHTC for couples in a relationship, the WHO comparison of CHTC versus individual HIV testing could be used to facilitate group discussion. In addition, an illustrative example of a serodiscordant couple could explain how a couple can have different test results over a long period of time, and therefore an infection does not automatically indicate infidelity during the relationship. In line with guidance for CHTC, discussions about dealing with HIV positive results could focus on the importance of planning for their future together as a couple, rather than considering the source of any potential infection in the past.

In order to increase opportunities for discussing barriers to CHTC, peer mentors from the local community could facilitate a dialogue about barriers to couples testing and share their reflections of testing for HIV while in a relationship and disclosing their status to their partner. This would provide information from a credible and relatable source, matching peer mentors and participants' gender to increase relevance to the participants. The community working group agreed that this would be an important addition to the intervention which could help encourage CHTC uptake through providing a role model and increasing understanding.

3.2.2 Optimisation to overcome fears about receiving an HIV positive diagnosis

Believing that treatment is both effective and accessible if needed, and that being HIV positive does not have to mean sickness and losing your status and respect within the community could help people perceive HIV testing as more worthwhile. The intervention could include additional

For peer review only - http://bmjopen.bmj.com/site/about/guidelines.xhtml

information about antiretroviral therapy availability and its benefits, and HIV positive peer mentors could reflect on how treatment helps keep them healthy.

3.2.3 Optimisation to promote benefits of open discussion in a single-gender group

Due to the value placed on single-gender sessions, the total time for participants in single gender sessions could be increased in order to facilitate an open dialogue around barriers to HIV testing and associated implications at an earlier point in the intervention.

3.2.4 Optimisation to overcome intervention facilitators providing advice to couples

Training facilitators in the value of open questioning could help enable a couple to discuss why they are not ready to test together, and to come up with their own solutions. This training would encourage facilitators that it is not their responsibility to ensure couples engage in CHTC, and that their success as facilitators is not evaluated based on CHTC uptake. The Healthy Conversations Skills Training package (17) was identified as an appropriate tool to deliver this message during facilitator training. This emphasises to facilitators that their role is to help people recognise their own barriers and to ask open discovery questions to facilitate them in identifying solutions.

The key barriers identified to CHTC in the Uthando Lwethu intervention and proposed optimisations are outlined in table 3.

<Insert table 3 here>

3.3 What are the theorised mechanisms of action through which the optimised intervention will change behaviour?

A logic model was developed to show how this optimised intervention is theorised to promote CHTC uptake (Figure 1). As in Uthando Lwethu, transformation of motivation remains a key mechanism through which the intervention seeks to increase CHTC uptake. This construct comes from communal coping theory (Lewis et al. 2006) which proposes that by improving relationship

For peer review only - http://bmjopen.bmj.com/site/about/guidelines.xhtml

For peer review only

functioning and communication within a couple, motivation for looking after health can be shifted from the individual to the couple, which would prompt more couples to engage in testing together in order to look after their partner's health as well as their own. In Uthando Lwethu, this approach informed the couples counselling sessions which aimed to improve relationship functioning and communication skills, thereby facilitating difficult discussions that partners may wish to have and indirectly influencing CHTC uptake.

The optimised intervention still seeks to promote transformation of motivation amongst couples, but alongside this it has a more direct focus on increasing understanding of the consequences of testing, changing outcome expectancies, and managing negative emotions around HIV testing. This was informed by the qualitative interviews which revealed that while many couples enjoyed the relationship counselling in Uthando Lwethu, often they did not discuss barriers to HIV testing with their partner but instead focused on other issues in their relationship, such as financial concerns. While addressing these issues can help to bring couples closer, the interviews suggested some people needed further support to feel ready to test for HIV. Therefore, the optimised intervention is theorised to operate both through an indirect pathway of improving relationship functioning and intimacy, but also through a more direct pathway of overcoming barriers to CHTC. Key differences from Uthando Lwethu are shown in red.

<Insert Figure 1 here>

4. Discussion

This study adopted the person-based approach to explore couples' and facilitators' experiences and perceptions of an intervention to promote CHTC uptake in KZN, South Africa. Suggested optimisations to the intervention were identified through developing an in-depth understanding of

For peer review only - http://bmjopen.bmj.com/site/about/guidelines.xhtml

people's beliefs and perceived barriers to couples testing for HIV. These included group discussion facilitated by peer mentors to help improve outcome expectancies about living with HIV and address concerns about the impact on relationships, a more explicit focus on discussing barriers to CHTC during couples' counselling sessions, and more time in single-gender sessions in order to facilitate open discussion about HIV.

The use of rigorous qualitative methods was a strength of this study as it provided an in-depth understanding of facilitators and barriers to couples' HIV testing, which enabled tailored optimisation of the intervention components. This is in line with recommendations that qualitative research be used to explore how HIV interventions might change behaviour to help enhance effectiveness (18). A limitation was that the interviews were conducted approximately five years after the intervention was delivered, although most people still had detailed recollections about their experiences. A challenge in conducting the qualitative interviews was that while participants openly discussed perceived barriers to CHTC, very few responded to questions about aspects of the intervention they disliked, preferring to answer that they were very happy with everything. Honest evaluation of an intervention by its users is very helpful for understanding people's needs and how best to overcome behavioural barriers (19), and therefore the interview schedule was adjusted to provide participants with more context about how their answers could help improve the intervention for future users. However, participants were still not forthcoming about anything they disliked or found more challenging. It is possible that the use of vignettes about a fictional person may have helped participants feel comfortable to provide more open feedback. This technique was found to successfully overcome social desirability bias and elicit feedback from participants which appeared to accurately reflect their own beliefs (20, 21). Consulting the community working group about the development of vignettes and conducting pilot interviews in the community to test the interview schedule might have helped address this issue. Meanwhile intervention staff were very reflective in their interviews and shared detailed insights into their experiences of delivering the intervention.

For peer review only - http://bmjopen.bmj.com/site/about/guidelines.xhtml

Implied lack of trust is widely recognised as a barrier to discussing and engaging in CHTC, as couples describe associating HIV testing with implications of recent infidelity (Parker et al 2014, Conroy 2014). This is theorised to be exacerbated by social norms for multiple partners and low marriage rates in South Africa, campaigns that associate HIV prevention with monogamy, and lack of knowledge about the potential for long-term differences in HIV status within couples represented by serodiscordance (such that HIV infection could have occurred a while ago, rather than indicating recent unfaithfulness) (10). This presents a significant challenge to implementation of CHTC, and supports the need for a supportive environment for couples to discuss HIV testing, with a focus on ensuring the best future together rather than considering past infidelities, as well as education about serodiscordance. The intervention optimisations proposed by this study are supported by the literature as effective strategies for facilitating CHTC uptake, including understanding the benefits of CHTC compared with individual testing, and peer mentors to facilitate discussions (22, 23). The study also offered novel insights into the experiences of counsellors delivering a couples-focused intervention for HIV testing. As asking open questions is known to be effective to support behaviour change, whilst telling or giving advice can lead to disagreement from the recipient (24), the findings from this study suggested that additional training for facilitators to support open questioning and absolve personal responsibility for couples' decisions may be beneficial.

The optimised intervention will be evaluated in a study in KZN and CHTC uptake will be compared with uptake in the intervention arm of the original RCT. Process interviews will be used to explore couples' experiences of the optimised intervention. The process of optimisation could be further streamlined in future research by adopting a flexible adaptive trial design which allows modifications to be made in line with ongoing qualitative process research, such that barriers can be addressed as they come to light (25).

For peer review only - http://bmjopen.bmj.com/site/about/guidelines.xhtml

5. Conclusions

In-depth qualitative interviews identified key barriers to engaging with CHTC, including concerns about trust in the relationship, and a lack of open discussion about barriers to CHTC during counselling sessions. This informed proposed optimisations to an existing couples-focused intervention in line with evidence and theory to overcome these barriers, including the addition of a discussion with peer mentors, education about serodiscordance, and training facilitators to use open questions in counselling sessions to explore couples' barriers to CHTC. The use of the person-based approach to intervention optimisation ensured that these changes were grounded in participants' experiences and unique psychosocial context.

For peer review only

For peer review only - http://bmjopen.bmj.com/site/about/guidelines.xhtml

Acknowledgements:

Thank you to all participants in this research study.

The following people have provided helpful comments or suggestions, or have otherwise enabled this work: Dr Tawanda Makusha (HSRC), and Prof Lynae Darbes (University of Michigan).

Funding:

This report is independent research supported by the National Institute for Health Research using Official Development Assistance (ODA) funding (NIHR Global Health Research Professorship, Professor Nuala McGrath, RP-2017-08-ST2-008). KM, TM, LM, NN, ZF and NMcG were supported by this funding. The views expressed in this publication are those of the author(s) and not necessarily those of the NHS, the National Institute for Health Research or the Department of Health and Social Care.

Competing interests: The authors have no competing interests to declare.

Authors' contributions: LM and TM conducted the interviews. KM trained the interviewers, conducted some staff interviews, analysed the data, and wrote the manuscript. AVH, TN, PJ, VH, HVR and NM contributed to the original intervention design and conducted the 'Uthando Lwethu' trial, as well as planning and organising the present qualitative study. NN coordinated the participant recruitment, data collection and community working group and participant feedback events. ZF contributed to intervention optimisation. All authors contributed to decisions about intervention optimisations and reviewed the manuscript.

Data sharing statement: The interview transcripts will not be made available to protect participants' anonymity.

For peer review only

For peer review only - http://bmjopen.bmj.com/site/about/guidelines.xhtml

References

1. World Health Organization. Guidance on couples HIV testing and counselling including antiretroviral therapy for treatment and prevention in serodiscordant couples: recommendations for a public health approach. 2012.
2. Kilembe W, Wall KM, Mokgoro M, Mwaanga A, Dissen E, Kamusoko M, et al. Implementation of couples' voluntary HIV counseling and testing services in Durban, South Africa. BMC Public Health. 2015;15(1):601.
3. Wall K, Allen S. Incentives to improve couples' HIV testing uptake and cost-effectiveness. The Lancet Global Health. 2017;5(9):e847-e8.
4. Vandormael A, Cuadros D, Kim HY, Barnighausen T, Tanser F. The state of the HIV epidemic in rural KwaZulu-Natal, South Africa: a novel application of disease metrics to assess trajectories and highlight areas for intervention. Int J Epidemiol. 2020.
5. Kim HY, Harling G, Vandormael A, Tomita A, Cuadros DF, Barnighausen T, et al. HIV seroconcordance among heterosexual couples in rural KwaZulu-Natal, South Africa: a population-based analysis. J Int AIDS Soc. 2020;23(1):e25432.
6. World Health Organization. Guideline on when to start antiretroviral therapy and on pre-exposure prophylaxis for HIV. 2015.
7. United Nations General Assembly. United Nations General Assembly. Political Declaration on HIV and AIDS: on the Fast-Track to accelerating the fight against HIV and to ending the AIDS epidemic by 2030. . New York, USA2016.
8. Darbes LA, McGrath NM, Hosegood V, Johnson MO, Fritz K, Ngubane T, et al. Results of a couples-based randomized controlled trial aimed to increase testing for HIV. JAIDS Journal of Acquired Immune Deficiency Syndromes. 2019;80(4):404-13.
9. Yardley L, Morrison L, Bradbury K, Muller I. The person-based approach to intervention development: application to digital health-related behavior change interventions. J Med Internet Res. 2015;17(1):e30.
10. Parker L, Pettifor A, Maman S, Sibeko J, MacPhail C. Concerns about partner infidelity are a barrier to adoption of HIV-prevention strategies among young South African couples. Cult Health Sex. 2014;16(7):792-805.
11. Conroy AA. 'It means there is doubt in the house': perceptions and experiences of HIV testing in rural Malawi. Culture, Health & Sexuality. 2014;16(4):397-411.
12. Pettifor A, MacPhail C, Nguyen N, Rosenberg M, Parker L, Sibeko J. Feasibility and acceptability of Project Connect: a couples-based HIV-risk reduction intervention among young couples in Johannesburg, South Africa. AIDS Care. 2014;26(4):476-82.
13. Duncan E, O'Cathain A, Rousseau N, Croot L, Sworn K, Turner KM, et al. Guidance for reporting intervention development studies in health research (GUIDED): an evidence-based consensus study. BMJ Open. 2020;10(4):e033516.
14. Darbes LA, van Rooyen H, Hosegood V, Ngubane T, Johnson MO, Fritz K, et al. Uthando Lwethu ('our love'): a protocol for a couples-based intervention to increase testing for HIV: a randomized controlled trial in rural KwaZulu-Natal, South Africa. Trials. 2014;15(1):64.
15. Markman HJ, Renick MJ, Floyd FJ, Stanley SM, Clements M. Preventing marital distress through communication and conflict management training: a 4- and 5-year follow-up. J Consult Clin Psychol. 1993;61(1):70-7.
16. Hosegood V, McGrath N, Moultrie T. Dispensing with marriage: Marital and partnership trends in rural KwaZulu-Natal, South Africa 2000-2006. Demographic Research. 2009;20:279-312.
17. Vogel C, Lawrence W, Cradock S, Ntani G, Tinati T, Jarman M, et al. Healthy Conversation Skills: increasing competence and confidence in front-line staff. Public Health Nutr. 2012;17:1-8.
18. Camlin CS, Seeley J. Qualitative research on community experiences in large HIV research trials: what have we learned? J Int AIDS Soc. 2018;21 Suppl 7:e25173.

For peer review only - http://bmjopen.bmj.com/site/about/guidelines.xhtml

19. Bradbury K, Morton K, Grist R, Band R, Van Woezik A, Connelly D, et al. Using the Person-Based Approach to optimise a digital intervention for the management of hypertension. PLoS One. 2018;13(5):e0196868.
20. Gourlay A, Mshana G, Birdthistle I, Bulugu G, Zaba B, Urassa M. Using vignettes in qualitative research to explore barriers and facilitating factors to the uptake of prevention of mother-to-child transmission services in rural Tanzania: a critical analysis. BMC Med Res Methodol. 2014;14(1):21.
21. Adanikin AI, McGrath N, Padmadas SS. Power relations and negotiations in contraceptive decision-making when husbands oppose family planning: analysis of ethnographic vignette couple data in Southwest Nigeria. Culture, health & sexuality. 2019:1-13.
22. Nannozi V, Wobudeya E, Matsiko N, Gahagan J. Motivators of couple HIV counseling and testing (CHCT) uptake in a rural setting in Uganda. BMC Public Health. 2017;17(1):104.
23. Adeomi AA, Adeoye OA, Asekun-Olarinmoye EO, Abodunrin OL, Olugbenga-Bello AI, Sabageh AO. Evaluation of the Effectiveness of Peer Education in Improving HIV Knowledge, Attitude, and Sexual Behaviours among In-School Adolescents in Osun State, Nigeria. AIDS Res Treat. 2014;2014:131756.
24. Mason P, Butler C. Health Behavior Change: A Guide For Practitioners2010.
25. Pallmann P, Bedding AW, Choodari-Oskooei B, Dimairo M, Flight L, Hampson LV, et al. Adaptive designs in clinical trials: why use them, and how to run and report them. BMC Med. 2018;16(1):29-.

For peer review only

Table 2. Guiding principles to inform the optimisation of the intervention

**Design objectives**	**Key intervention features**
Help couples feel close to each other, recognising the positive aspects of their relationship and providing motivation to look after their health together.	- Activities to promote focus on the positive aspects of the relationship.
Encourage effective communication to address fears and outcome expectancies about testing.<br><br>Address negative emotions.	- Ask open questions to enable couples to identify their barriers to CHTC and come up with their own solutions.<br>- Provide effective communication skills training to help couples explore their perceived barriers to testing together.
Increase understanding about risk of HIV transmission, serodiscordance, and effectiveness of treatment.	- Interactive single-gender group activities<br>- Open dialogue facilitated by a peer mentor with experience of couples HIV testing<br>- Provide standard guidance on HIV prevention to reduce risk of transmission, e.g. condom demonstration.

For peer review only - http://bmjopen.bmj.com/site/about/guidelines.xhtml

Table 3. Key barriers or facilitators identified to CHTC and proposed optimisations

**Key barriers/facilitators**	**Optimisations to overcome the barriers**
Concerns about implications of CHTC for the relationship	Additional focused discussion about barriers to CHTC, dialogue facilitated by public speakers with experience of HIV testing, and education about serodiscordance to highlight that an HIV positive result in one partner does not automatically mean a recent infidelity.
Fear about finding out you are HIV positive	Education and discussion about the effectiveness and accessibility of ART.
Benefits of open discussion in single-gender group	Create more time in single-gender group sessions as both staff and participants felt these facilitated open discussion.
Intervention facilitators providing advice to couples	Tailored training for intervention facilitators in the value of open questions and enabling couples to come up with their own solutions to their barriers.

For peer review only

Figure 1. Optimised intervention logic model

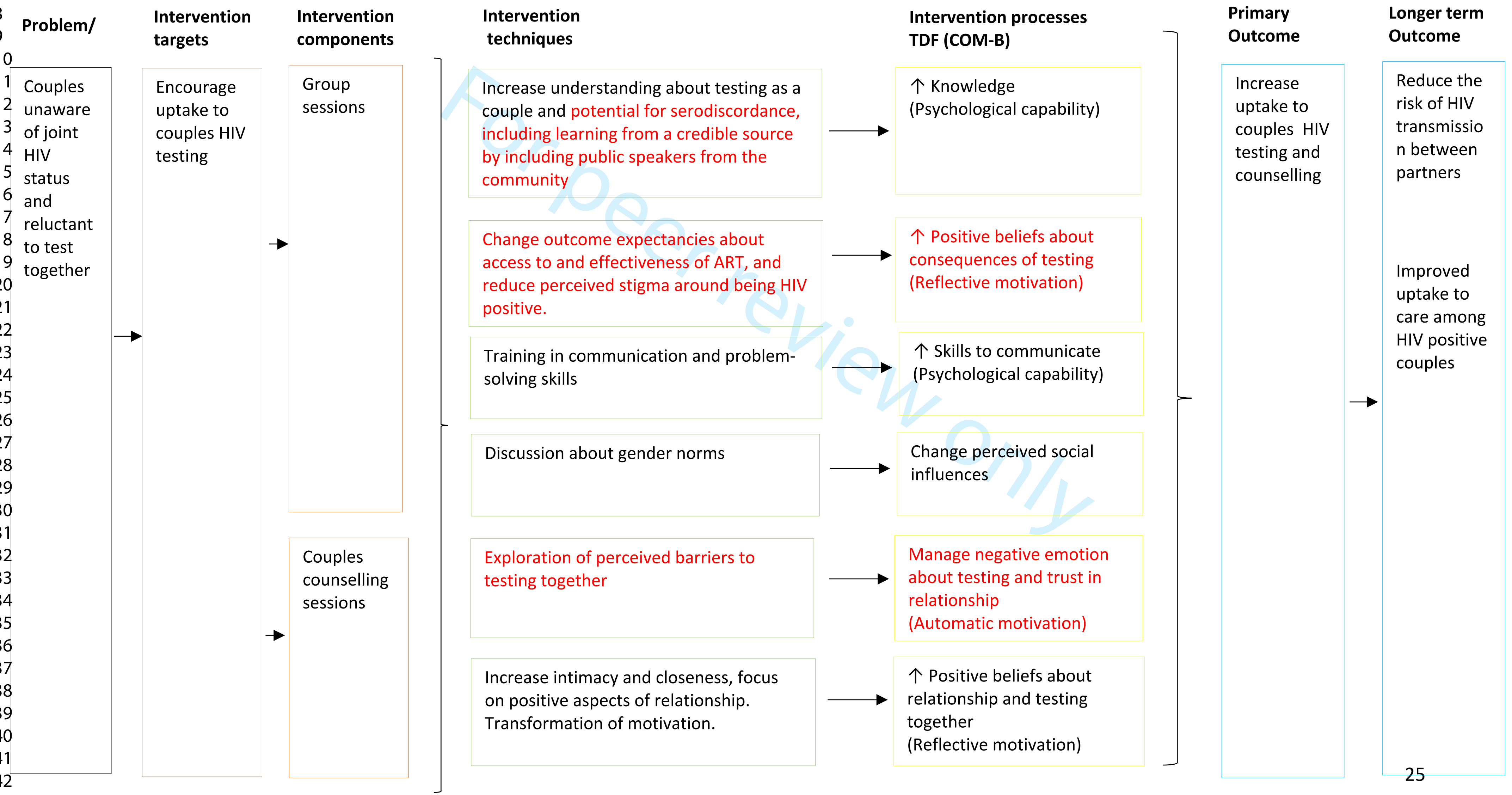

For peer review only - http://bmjopen.bmj.com/site/about/guidelines.xhtml

Consolidated criteria for reporting qualitative studies (COREQ): 32-item checklist

Developed from:
Tong A, Sainsbury P, Craig J. Consolidated criteria for reporting qualitative research (COREQ): a 32-item checklist for interviews and focus groups. *International Journal for Quality in Health Care*. 2007. Volume 19, Number 6: pp. 349 – 357

YOU MUST PROVIDE A RESPONSE FOR ALL ITEMS. ENTER N/A IF NOT APPLICABLE

No. Item	Guide questions/description	Reported on Page #
**Domain 1: Research team and reflexivity**		
Personal Characteristics		
1. Interviewer/facilitator	Which author/s conducted the interview or focus group?	Page 9 and 10
2. Credentials	What were the researcher's credentials? E.g. PhD, MD	Page 9 and 10
3. Occupation	What was their occupation at the time of the study?	Page 9 and 10
4. Gender	Was the researcher male or female?	Page 9 and 10
5. Experience and training	What experience or training did the researcher have?	Page 9
Relationship with participants		
6. Relationship established	Was a relationship established prior to study commencement?	8
7. Participant knowledge of the interviewer	What did the participants know about the researcher? e.g. personal goals, reasons for doing the research	Supplementary Files 2 and 3
8. Interviewer characteristics	What characteristics were reported about the interviewer/facilitator? e.g. Bias, assumptions, reasons and interests in the research topic	Supplementary Files 2 and 3
**Domain 2: study design**		
Theoretical framework		
9. Methodological orientation and Theory	What methodological orientation was stated to underpin the study? e.g. grounded theory, discourse analysis, ethnography, phenomenology, content analysis	Page 7 and 10
Participant selection		
10. Sampling	How were participants selected? e.g. purposive, convenience, consecutive, snowball	Page 8
11. Method of approach	How were participants approached? e.g. face-to-face, telephone, mail, email	Page 9

For peer review only

12. Sample size	How many participants were in the study?	Page 11
13. Non-participation	How many people refused to participate or dropped out? Reasons?	Page 9
Setting		
14. Setting of data collection	Where was the data collected? e.g. home, clinic, workplace	Page 9
15. Presence of non-participants	Was anyone else present besides the participants and researchers?	N/A
16. Description of sample	What are the important characteristics of the sample? e.g. demographic data, date	Page 11
Data collection		
17. Interview guide	Were questions, prompts, guides provided by the authors? Was it pilot tested?	Supplementary Files 2 and 3
18. Repeat interviews	Were repeat inter views carried out? If yes, how many?	N/A
19. Audio/visual recording	Did the research use audio or visual recording to collect the data?	Page 9
20. Field notes	Were field notes made during and/or after the inter view or focus group?	Page 9
21. Duration	What was the duration of the inter views or focus group?	Page 11
22. Data saturation	Was data saturation discussed?	N/A
23. Transcripts returned	Were transcripts returned to participants for comment and/or correction?	N/A
**Domain 3: analysis and findings**		
Data analysis		
24. Number of data coders	How many data coders coded the data?	Page 10
25. Description of the coding tree	Did authors provide a description of the coding tree?	N/A
26. Derivation of themes	Were themes identified in advance or derived from the data?	N/A
27. Software	What software, if applicable, was used to manage the data?	N/A
28. Participant checking	Did participants provide feedback on the findings?	Page 10
Reporting		
29. Quotations presented	Were participant quotations presented to illustrate the themes/findings? Was each quotation identified? e.g. participant number	Page 12-14
30. Data and findings consistent	Was there consistency between the data presented and the findings?	Yes
31. Clarity of major themes	Were major themes clearly presented in the findings?	Yes
32. Clarity of minor themes	Is there a description of diverse cases or discussion of minor themes?	Yes

For peer review only

Once you have completed this checklist, please save a copy and upload it as part of your submission. When requested to do so as part of the upload process, please select the file type: *Checklist*. You will NOT be able to proceed with submission unless the checklist has been uploaded. Please DO NOT include this checklist as part of the main manuscript document. It must be uploaded as a separate file.

For peer review only

Supplementary File 2: Semi-structured interview schedule for participants in the couples-focused intervention

A. Introduction and thanks
My name is ************ and I am working for the 'Egugu Lethu project' which is being conducted by the Human Sciences Research Council and the University of Southampton, UK.<br><br>First, let me thank you for agreeing to speak to me today.

B. Project explanations
The overall aim of the 'Egugu Lethu project is to improve the couples counselling intervention that we used in the Uthando Lwethu study and to conduct a new study to test the improved intervention success in promoting couples HIV testing and counselling.<br><br>This interview is part of the first steps towards improving the previously tested intervention. We are conducting interviews with individual members of couples from the Uthando Lwethu study to explore their experiences of the intervention and it's role, if any, in contributing to their decision regarding uptake of couples HIV testing and counselling.

Interview explanations

Today I would like to ask you about your experiences of participating in the Uthando Lwethu Study. We would really like to hear your experiences, opinions and observations. I will ask you a series of questions from my guideline, but please feel free to add in extra comments that you think are important.
[I will be taping / I will not be taping] this interview and taking a few notes to aid my memory as we go along but I will not write down your name or the name of your partner.
I will not be using a questionnaire but have a list of topics that I'd like us to try and cover today. Think of our session as a conversation and that you can ask me questions too.

Great, so now we have completed the formalities, is it fine with you that we start?

What is your name?

And the name of your partner who participated with you in Uthando Lwethu Study?

General experience of the Uthando Lwethu Study

Before we start talking in detail about your experiences of specific parts of the Uthando Lwethu Study, can you tell me about the first day when you were approached about the study? What was that like for you?
Can you remember why you decided to sign up for the study?

For peer review only - http://bmjopen.bmj.com/site/about/guidelines.xhtml

I'm really interested in hearing about your experiences of the Uthando Lwethu study, Can you tell me what you remember about being in the study?

How did you find the sessions?

Does anything stand out in your memory about the study? What do you remember and why?

Can you tell me about what difference Uthando Lwethu made for you, if any?

Experience of the first group session

As a quick reminder, the first group session was where we gave some information about health to the group of couples attending together and at the end we had the lottery where you learned you and your partner were going to receive the Uthando Lwethu couples counselling sessions .

Can you tell me all about what the first group session was like for you?

[Note to interviewer: information given was on TB, reproductive health/HIV), and a list of community resources for health care and treatment was given]

What information did you find most helpful? And why?

What other information would you have liked?

How was it to see other couples from your area at the same session? Did you know anyone else there? Who?

Can you remember how you sat in the first session? Did you sit with your partner, or with other men/women?

How did you interact with other couples from your area?

Can you tell us about anything you remember learning in the first session?

Experience of the second group session

The second group session was where you met in [men-only / female only] groups and discussed roles, violence and power within relationships. There was also skills training for communication and risk reduction. What was your experience of that second group session?

What was most helpful in this session? Why?

What other information would you have liked? Why?

For peer review only - http://bmjopen.bmj.com/site/about/guidelines.xhtml

Can you tell me about any conversations you had with your partner about that session afterwards?

What did you discuss? Once off or repeatedly?

Were there any particular topics that you discussed in particular? [prompt for specific exercises / content that was included in the session to help participants remember]

Was one same gender group session enough or would you have liked more? Why?

Couples sessions

The next section is dependent on whether they attended the couples' sessions. Only one block of questions from this section is applicable in any particular individual interview.

a. For those who did NOT attend couples sessions at all

If you remember, you were then offered 4 couples' sessions *with* <counsellor's name> Can you tell me about why you did not attend the couples' sessions that were on offer?

How did you reach that decision?

Was this your choice or your partner's, or mutual?

What were your main concerns about the couples' sessions?

Could the study have done something differently that might have changed your mind? Or your partner's mind? Or made it possible logistically?

b. For those who attended one or more couples sessions

What was your main reason for deciding to attend the couples' sessions?

How did you reach that decision?

Did you have any concerns or worries about attending?

Do you remember your counsellor? [check they mean the same person recorded on page 1]. I am interested in how you felt about the couples' sessions that you attended.

If I asked you to describe ~~them~~ *the sessions in one or two sentences*, what would you say?

For peer review only - http://bmjopen.bmj.com/site/about/guidelines.xhtml

*Did you feel you were able to communicate your point of view and thoughts to your **partner** in the counselling session? And did your partner? Can you tell me about why?*

*Did you feel you were able to communicate your point of view and thoughts to the **counselor** in the counselling session? Can you tell me about why?*

How do you think your partner felt about it?

Was there anything you would change?

***[If they used all 4 sessions]:** Was the number of sessions enough*

***[If they used less than 4 sessions]:** Can you tell me about why you stopped attending when you did?*

Would having an additional session made a difference? Was another session likely to have changed your decision about testing?

Can you tell us about anything you remember learning from the sessions? [prompt for specific tasks or exercises / content to help participants remember]

Did you discuss anything about the session or from a session with your partner afterwards? What did you talk about?

Do you remember some of the homework exercises that the counsellor gave you? How did you and your partner get on with the homework?

(If still together) Can you tell me how you feel about communicating with your partner now?

Are you still practicing the skills taught? Why or why not? If not, how long after did you stop using the skills?

Cohabitation

The next section is dependent on the couple cohabitation status at the time of their participation in the Uthando Lwethu study. Only one block of questions from this section is applicable in any particular individual interview.

a. For those cohabiting at the time

At the time of the Uthando Lwethu study, you were living with your partner.

Can you tell me about what it was like doing the Uthando Lwethu study while you were living at home with your partner?

Did you want to talk to your partner about Uthando Lwethu?

If yes – did you have the opportunity to talk to them at home?

If no – why not?

Was anything different at home when you were both doing Uthando Lwethu?

For peer review only - http://bmjopen.bmj.com/site/about/guidelines.xhtml

b. For those NOT cohabiting at the time

At the time of the Uthando Lwethu study, you were not living with your partner.

Can you tell me about what it was like doing the Uthando Lwethu study while you were living separately from your partner?

Did you want to talk to your partner about Uthando Lwethu?

If yes – did you have the opportunity to talk to them? Where did the conversations happen?

If no – why not?

Was anything different when you met up while you were both doing Uthando Lwethu?

HIV testing

The next section is for ALL individuals

What do you think about HIV testing?

And testing with a partner together?

What are your main concerns about HIV testing?

How could we address those concerns?

The next section is dependent on whether the couple tested for HIV as a couple during the Uthando Lwethu study. <u>Only one block</u> of questions from this section is applicable in any particular individual interview.

a. For those who did not test for HIV as a couple during the Uthando Lwethu Study

I'm really interested in your decision around whether to test for HIV with your partner. It looks like you decided not to test together during the study, can you tell me more about that decision?

Did you as a couple discuss testing? Why or why not? How did you reach a decision?
How did you feel about getting tested?

How did your partner feel?

For peer review only - http://bmjopen.bmj.com/site/about/guidelines.xhtml

What were your main concerns? What could be done to address those concerns? Eg more information? sessions? support?

What do you think might have been the benefits of testing for you?

How did you resolve any conflict with your partner?

Did you test together anytime after the study had finished? How long after the study?

Do you know your partner's status? How do you know this?

Do you think the group sessions or couples counseling sessions contributed to your / your partner's decision?

b. For those who tested for HIV as a couple during the Uthando Lwethu Study

Have you ever tested together before Uthando Lwethu?
If yes - how did you decide to do this?

Can you tell me about why you decided to test together during the Uthando Lwethu study?

How did you reach a decision?

How did you feel about getting tested?

How did your partner feel?
Did you have any concerns about the testing? What could be done to address those concerns? Eg more information? sessions? support?

How did you resolve any conflict with your partner about deciding whether to get tested?

How did you arrange the testing?

How did you feel after the testing?

Did you talk about it with your partner? What do you think were the benefits of testing for you?

How would you encourage other couples to have HIV testing?

For peer review only - http://bmjopen.bmj.com/site/about/guidelines.xhtml

Do you think the group sessions or couples counseling sessions contributed to your / your partner's decision?

If yes: What part of the intervention encouraged you to be tested?

If not, why not?

For all individuals

In the new study, we may offer testing for sexually transmitted infections (STIs), specifically testing for chlamydia, syphilis, gonorrhoea and trichomoniasis. However we don't know if this would be useful for people, or what some of the concerns might be in having STI testing.

Can you tell me what you think about the idea of testing couples for sexually transmitted infections?

If we had also invited you to consider STI testing as part of the couples HIV testing session, how would you have felt about that?

Would you have taken up an offer for STI testing? Why/why not?

The STI testing would mean giving a sample that would be sent away to the labs for testing and you and your partner would come back to receive the results, For men, this would involve providing a urine sample. How would you feel about that?

For women, this would mean privately taking a vaginal swab. Many women do this themselves, or the nurse could help you with if you wanted. How would you feel about that?

Overall, what do you think about the Uthando Lwethu study for other people?

We are hoping to make some changes to Uthando Lwethu to make it even better for people. Can you think of any other health or relationship topics you would have liked to know more about in group session 1, group session 2, or the counselling sessions?

Can you think of anything that would have made Uthando Lwethu better for you?

And was there any part of Uthando Lwethu that you think was not needed or was not helpful to you?

For peer review only - http://bmjopen.bmj.com/site/about/guidelines.xhtml

Supplementary File 3. Semi-structured interview schedule for staff members.

A. Introduction and thanks
My name is ************ and I am working for the 'Egugu Lethu project' which is being conducted by the Human Sciences Research Council and the University of Southampton, UK.<br><br>First, let me thank you for agreeing to speak to me today.
**B. Project explanations**
The overall aim of the Egugu Lethu project is to improve the couples counselling intervention that we used in the Uthando Lwethu study and to conduct a new study to test the improved intervention success in promoting couples HIV testing and counselling.<br><br>The first step of optimising the previously tested intervention is to conduct interviews with individual members of couples from the Uthando Lwethu study and with staff members who were involved in delivering the intervention components. In staff interviews we will discuss staff experiences of the previous study and possible barriers to couples' decisions to test for HIV together.

Interview explanations

Today I would like to ask you about your experiences as an Uthando Lwethu Study staff member. We would really like to hear your experiences, opinions and observations. I will ask you a series of questions from my guideline, but please feel free to add in extra comments that you think are important. [I will be taping / I will not be taping] this interview and taking a few notes to aid my memory as we go along but I will not write down your name. I will not be using a questionnaire but have a list of topics that I'd like us to try and cover today. Think of our session as a conversation and that you can ask me questions too.

Great, so now we have completed the formalities, is it fine with you that we start?

What is your name?

What was your role in the Uthando Lwethu Study: Couples counsellor or part of the group sessions or counsellor for the HIV testing and counselling?

Can you tell me a bit about your background? I'd like to understand what your job is outside of Uthando Lwethu?

Can you tell me about the training you've had previously?

What was different in Uthando Lwethu from your usual counselling/facilitating role?

What was the same?

General experience of the Uthando Lwethu Study

For peer review only - http://bmjopen.bmj.com/site/about/guidelines.xhtml

Before we start talking in detail about your experiences and perceptions of specific parts of the Uthando Lwethu Study, I would like to hear what was it like being part of the study team overall?

How did you find the training you received before becoming a facilitator/counsellor for Uthando Lwethu?

The next section is dependent on which role the staff member had. Only one block of questions from this section is applicable in any particular individual interview.

For staff who attended group sessions

How many first group sessions did you attend? What was your role in the first group sessions?

what was your role in the second group sessions?

We're interested in how to improve the intervention for couples, so the next few questions are to try and find out more about how it worked last time, and what we could do better going forwards.

How did you find the first group sessions?

How did you find the second group sessions?

Did you notice any differences between the two sessions?

Can you recall what happened in a typical second group session?

What was it like following the manual for Uthando Lwethu during the second group session?

Were there any activities that stood out for you as being particularly important in the second group session?

Were there any activities that you remember being challenging for some people in the second group session?

How engaged did you feel people were? Can you tell me a story about any memorable moments you recall?

Are there any particular benefits/negatives to the single gender session?

For peer review only - http://bmjopen.bmj.com/site/about/guidelines.xhtml

How did the single gender sessions vary between groups?

What was it like to deliver this information to females/males as a man/woman yourself? Did the same / different gender dynamic work well or not really?

Could session 2 be reinforced by homework If we introduced homework at this session? do you think it would work best done as a couple of individually?

Did you feel there were any differences in the level of participation or questions between sessions?

How important do you think these group sessions were for encouraging couples to attend HIV testing together?

Do you think there was anything about the first or second group session that didn't work very well, that you think we should change for the next study? *[prompt for organizational or staff changes vs content]*

If **not** already mentioned, ask about:

In the first group session, how do you think couples found the information on:

- TB
- HIV
- HIV testing
- Contraception
- Alcohol

In the second group session, how do you think participants found the:

- Discussion around long-term relationships
- Condom demonstration
- Discussions about gender and power imbalance
- Practising communication skills

In the new study, we may offer testing for sexually transmitted infections (STIs), specifically testing for chlamydia, syphilis, gonorrhoea and trichomoniasis. Would any changes be needed in the way Group sessions 1 and 2 are conducted or their content if STI testing were offered alongside HIV testing and counselling?

How do you think couples would feel about testing for STIs together?
For men, it would involve providing a urine sample, and for women it would mean taking a vaginal swab. The samples are sent off to a lab, so the couple would need to come back to receive their results. How do you think this would work for couples in Uthando Lwethu?

For staff who conducted couples counselling sessions

For peer review only - http://bmjopen.bmj.com/site/about/guidelines.xhtml

Approximately, how many couples did you counsel?

How did you feel about the couples' counselling sessions that you led?

How do you think they were experienced by the participating couples?

Within those counselling sessions, how did you find it interacting with the female partner, as a female/male counsellor? And what about working with the male partner? Why?

Did the interaction with different couples vary? In what way did it vary and why?

Can you tell me about any reasons you can think of why some couples got more out of the counselling than others?

How do you think couples found the number of counselling sessions? Do you think having an additional session would make a difference / likely to have changed couples' decisions about testing?

How do you think the couples felt about practicing the speaker-listener technique between sessions?

Which exercises in the counselling sessions were particularly helpful for couples?

Were there any activities in the counselling sessions that you remember being challenging for some couples?

What was it like following the intervention manual in the couples counselling session?

If **not** already mentioned, ask the following:
In the couples counselling sessions, what do you think it was like for couples:

- Identifying and talking about their expectations and barriers around HIV testing
- Practising the speaker listener technique with their partner
- Planning solutions to problems together
- Setting some personal goals
- Doing the 'forgetting something good' activity, where they noticed what helpful things their partner has done for them recently
- Doing the 'words of the heart' activity where couples chose words that describe their relationship

If the participant only talks about positive examples where couples liked these things, ask:

- *Were there any couples who struggled to do that?*

For peer review only - http://bmjopen.bmj.com/site/about/guidelines.xhtml

- *If so, what was that like for you?*

Why do you think some couples didn't go for HIV testing? What do you think were the barriers for them?

Can you tell me about a couple who did go for testing? Why do you think they decided to get tested?

In the new study, we may offer testing for sexually transmitted infections (STIs), specifically testing for chlamydia, syphilis, gonorrhoea and trichomoniasis. Would any changes be needed in the way the couples counselling sessions are conducted or their content if STI testing were offered alongside HIV testing and counselling?

How do you think couples would feel about testing for STIs together?
For men, it would involve providing a urine sample, and for women it would mean taking a vaginal swab. The samples are sent off to a lab, so the couple would need to come back to receive their results. How do you think this would work for couples in Uthando Lwethu?

For peer review only

For peer review only - http://bmjopen.bmj.com/site/about/guidelines.xhtml

For staff who conducted couples HIV testing and counselling during the Uthando Lwethu Study

Approximately how many couples HIV testing and counselling sessions did you conduct during Uthando Lwethu Study?

How did you feel about the couples' HIV testing and counselling sessions that you conducted?

Did the couples discuss with you how they came to the decision to test for HIV together? What reason(s) did they give?

In the new study, we may offer testing for sexually transmitted infections (STIs), specifically testing for chlamydia, syphilis, gonorrhoea and trichomoniasisdo you think offering STI testing as part of the session would change couples' interest in testing for HIV together?

> *To what extent do you think including STI testing in the couples testing session would change this session for you as counsellor?*
>
> *How do you think couples would feel about testing for STIs together? For men, it would involve providing a urine sample, and for women it would mean taking a vaginal swab. The samples are sent off to a lab, so the couple would need to come back to receive their results. How do you think this would work for couples in Uthando Lwethu?*

For all staff at the end:

> *Did you have any contact with couples after **their** participation in the study had ended?*
>
> *What was the nature of those interactions? [Probe for friendly / casual, neighbour, asking advice, sought out staff member's skill as counsellor etc.]*

For peer review only

For peer review only - http://bmjopen.bmj.com/site/about/guidelines.xhtml

BMJ Open

Optimising a couples-focused intervention to increase couples' HIV testing and counselling using the person-based approach: A qualitative study in Kwa-Zulu Natal, South Africa.

Journal:	*BMJ Open*
Manuscript ID	bmjopen-2020-047408.R1
Article Type:	Original research
Date Submitted by the Author:	29-Mar-2021
Complete List of Authors:	Morton, Katherine; University of Southampton, Psychology<br>Mhlakwaphalwa, Tembeka; Human Sciences Research Council<br>Msimango, Lindani; Human Sciences Research Council<br>van Heerden , Alastair ; Human Sciences Research Council; University of the Witwatersrand Faculty of Health Sciences, School of Clinical Medicine<br>Ngubane, Thulani; Human Sciences Research Council<br>Joseph, Philip; Human Sciences Research Council<br>Ngcobo, Nathi; Human Sciences Research Council<br>Feng, Z; University of Southampton, Primary Care and Population Sciences<br>Hosegood, Victoria; University of Southampton, Department of Social Statistics and Demography, Faculty of Social Sciences<br>van Rooyen, Heidi; Human Sciences Research Council; University of the Witwatersrand<br>McGrath, Nuala; University of Southampton, School of Primary Care, Population Sciences and Medical Education, Faculty of Medicine; University of Southampton, Department of Social Statistics & Demography, Faculty of Social Sciences
**Primary Subject Heading**:	Sexual health
Secondary Subject Heading:	Public health, Qualitative research, HIV/AIDS
Keywords:	HIV & AIDS < INFECTIOUS DISEASES, PUBLIC HEALTH, QUALITATIVE RESEARCH

SCHOLARONE™
Manuscripts

I, the Submitting Author has the right to grant and does grant on behalf of all authors of the Work (as defined in the below author licence), an exclusive licence and/or a non-exclusive licence for contributions from authors who are: i) UK Crown employees; ii) where BMJ has agreed a CC-BY licence shall apply, and/or iii) in accordance with the terms applicable for US Federal Government officers or employees acting as part of their official duties; on a worldwide, perpetual, irrevocable, royalty-free basis to BMJ Publishing Group Ltd ("BMJ") its licensees and where the relevant Journal is co-owned by BMJ to the co-owners of the Journal, to publish the Work in this journal and any other BMJ products and to exploit all rights, as set out in our licence.

The Submitting Author accepts and understands that any supply made under these terms is made by BMJ to the Submitting Author unless you are acting as an employee on behalf of your employer or a postgraduate student of an affiliated institution which is paying any applicable article publishing charge ("APC") for Open Access articles. Where the Submitting Author wishes to make the Work available on an Open Access basis (and intends to pay the relevant APC), the terms of reuse of such Open Access shall be governed by a Creative Commons licence – details of these licences and which Creative Commons licence will apply to this Work are set out in our licence referred to above.

Other than as permitted in any relevant BMJ Author's Self Archiving Policies, I confirm this Work has not been accepted for publication elsewhere, is not being considered for publication elsewhere and does not duplicate material already published. I confirm all authors consent to publication of this Work and authorise the granting of this licence.

For peer review only

Optimising a couples-focused intervention to increase couples' HIV testing and counselling using the person-based approach: A qualitative study in Kwa-Zulu Natal, South Africa.

Kate Morton*[1] k.s.morton@soton.ac.uk

Tembeka Mhlakwaphalwa[2] tembekam23@gmail.com

Lindani Msimango[2] LIMsimango@hsrc.ac.za

Alastair Van Heerden[2, 3] avanheerden@hsrc.ac.za

Thulani Ngubane[2] tngubane@hsrc.ac.za

Phillip Joseph[2] pjoseph@hsrc.ac.za

Nathi Ngcobo[2] NNgcobo@hsrc.ac.za

Zhixin Feng[4] Z.Feng@soton.ac.uk

Victoria Hosegood[5] V.Hosegood@soton.ac.uk

Heidi Van Rooyen[2,3] hvanrooyen@hsrc.ac.za

Nuala McGrath[4,5] N.McGrath@soton.ac.uk

1. School of Psychology, University of Southampton, UK.

2. Human Sciences Research Council, Sweetwaters, KwaZulu-Natal, South Africa.

3. SAMRC/WITS Developmental Pathways for Health Research Unit Department of Paediatrics, School of Clinical Medicine, Faculty of Health Sciences, University of the Witwatersrand, Johannesburg, South Africa.

For peer review only

For peer review only - http://bmjopen.bmj.com/site/about/guidelines.xhtml

4. School of Primary Care, Population Sciences and Medical Education, Faculty of Medicine, University of Southampton, UK

5. Department of Social Statistics & Demography, Faculty of Social Sciences, University of Southampton, UK

*Corresponding author: k.s.morton@soton.ac.uk; Centre for Clinical and Community Applications of Health Psychology, B44, University of Southampton, Southampton, SO17 1BJ

For peer review only

For peer review only - http://bmjopen.bmj.com/site/about/guidelines.xhtml

Abstract:

Objectives: This qualitative study explored how to optimise a couples-focused intervention to promote couples HIV testing and counselling (CHTC).

Setting: Community setting in Kwa-Zulu Natal, South Africa.

Participants: Qualitative interviews were conducted with 20 couples who had participated in a couples-focused intervention and five staff members delivering the intervention. Partners were interviewed individually by researchers of the same gender.

Intervention: A couples-focused intervention comprised of two group sessions and four couples counselling sessions was previously shown to significantly increase uptake to CHTC in Kwa-Zulu Natal, South Africa. However, more than half of couples participating in the intervention still chose not to test together during follow-up.

Analysis: The transcripts were analysed using the table of changes from the person-based approach. Proposed optimisations were discussed with a community group to ensure the intervention was as persuasive and acceptable as possible.

Results: Many couples found it challenging to discuss CHTC with their partner due to an implied lack of trust. Optimisations to the intervention were identified to increase readiness to discuss CHTC, including education about serodiscordance, discussions about CHTC by peer mentors, and open discussion of personal barriers to CHTC during couples' counselling sessions. Additional training for staff in open questioning techniques could help them feel more comfortable to explore couples' perceived barriers to CHTC, rather than advising couples to test. A logic model was developed to show anticipated mechanisms through which the optimised intervention would increase uptake to

For peer review only - http://bmjopen.bmj.com/site/about/guidelines.xhtml

CHTC, including increasing knowledge, increasing positive outcome beliefs, and managing negative emotions.

Conclusions: In-depth qualitative research informed optimisations to a couples-focused intervention for further evaluation in South Africa to encourage uptake to CHTC. Suggestions are made for optimal methods to gain open feedback on intervention experiences where participants may be reluctant to share negative views.

Key words: Couples-focused, intervention, Couples HIV Counselling and Testing, person-based approach

For peer review only

For peer review only - http://bmjopen.bmj.com/site/about/guidelines.xhtml

Strengths and Limitations of this study

- Conducting qualitative interviews with partners separately, in their own homes, and with a researcher of the same gender helped promote rapport and facilitated open reflections about their relationship.
- Interviewing staff members responsible for delivering the intervention in addition to couples participating in the intervention provided a more in-depth understanding of potential barriers to CHTC.
- The Table of Changes from the Person-Based Approach provided a rigorous method to analyse the qualitative interviews. The use of guiding principles and a logic model ensured that optimisations were grounded in the specific psychosocial context of this population and drew on theoretical constructs.
- Conducting the interviews five years after the intervention had been delivered may have limited participants' ability to accurately recall their experiences and perceptions.
- Many participants appeared reluctant to share any negative views of the intervention, and alternative methods such as the use of vignettes may have helped obtain more critical feedback about the couples-focused intervention.

For peer review only

For peer review only - http://bmjopen.bmj.com/site/about/guidelines.xhtml

1. Introduction

Couples HIV Testing and Counselling (CHTC) is recommended by the World Health Organisation (1) and is now part of South African policy guidelines (2), however, very few couples in South Africa have tested together (3, 4). This is against a background of continued high levels of HIV prevalence, particularly in KwaZulu-Natal (KZN), South Africa. In a large community-based survey of adults in KZN, 19% of men and 41% of women were living with HIV in 2017 (5). The majority of couples in stable sexual relationships reported being unaware of their partner's HIV status (57%) (6), and testing rates remain low, particularly for men (7). CHTC can contribute to reductions in HIV incidence as a compliment to the universal test and treat policy in South Africa (8) and individual focused testing campaigns (9).

In 2012-2015, a couples-focused intervention ('Uthando Lwethu'*)* was implemented in a rural community setting in uMgungundlovu, KZN. This community has high levels of migration and unemployment, and low marriages rates, so many couples in the intervention were neither married nor cohabiting (10). At the outset, nearly 40% of the sample had never had an HIV test, and most had not shared their HIV status with their current partner (10). The intervention was shown to significantly increase uptake to CHTC, with 42% of couples attending CHTC within a 9-month follow-up period compared with 12% in the control group (10). Nonetheless, over half of couples receiving the intervention chose not to test within the study period.

In order to optimise the couples-focused intervention for future implementation, we undertook further supplementary research to explore how the intervention content and delivery could be modified to support couples to feel ready for CHTC. This paper presents the findings from a qualitative study with couples and staff involved in the Randomised Controlled Trial (RCT) of the Uthando Lwethu intervention. The person-based approach (PBA) (Yardley et al. 2015) was adopted, which complements evidence and theory in developing and optimising effective interventions and

For peer review only - http://bmjopen.bmj.com/site/about/guidelines.xhtml

has been used to successfully create behaviour change interventions that are feasible, persuasive, motivating and engaging (11). This approach recommends conducting in-depth research to develop an understanding of the psychosocial context and underlying beliefs in the target population that could influence engagement with behaviour change. Guiding principles are developed to describe specific behavioural barriers for a given population and context and identify particular intervention features to overcome these barriers. In addition, this intervention optimisation process was informed by recent evidence regarding facilitators to CHTC (12-14), and a consideration of the theoretical mechanisms through which the optimised intervention might impact couples' readiness to undertake CHTC.

Our research questions were:

a. How can a couples-focused intervention be optimised to overcome barriers to engaging in CHTC in KwaZulu-Natal?
b. What are the theorised mechanisms of action through which the optimised intervention is anticipated to change behaviour?

2. Methods

2.1 Optimisation study design

Figure 1 shows how this optimisation study fitted into a wider body of research, with the orange box showing the present study.

<Insert Figure 1>

This was a qualitative study using retrospective semi-structured interviews to explore possible barriers to engaging in CHTC amongst couples participating in the Uthando Lwethu intervention. Due to the logistics of research funding and contracts, the interviews were conducted approximately five years after participants took part in the intervention. We report the optimisation process using the

For peer review only - http://bmjopen.bmj.com/site/about/guidelines.xhtml

GUIDED checklist (Guidance for reporting intervention development studies in health research) in line with best practice for reporting intervention development (15). The COREQ checklist was used for reporting (Supplementary File 1). Approval was granted by the Human Sciences Research Council (REC 2/19/10/11b) and the University of Southampton (reference number: ERGO 48702) ethics committees.

2.2 Uthando Lwethu Intervention, prior to optimisation

As this study aimed to describe the optimisation of a couples-focused intervention to promote CHTC, the original content and delivery of the Uthando Lwethu intervention trialled in the 2012-2015 randomised controlled trial (16) is described here.

Uthando Lwethu was a couples-focused intervention comprised of two group sessions and four couples counselling sessions that aimed to increase CHTC uptake. It was developed based on interdependence theory which states that positive relationship dynamics can lead to a transformation of motivation, in which couples are motivated to engage in health behaviours due to a focus on the relationship rather than an individual perspective. It was theorised that couples would be more willing to engage in CHTC if they have greater commitment and trust in their relationship (16). The intervention was adapted from the Prevention and Relationship Enhancement Programme (PREP), which aims to improve relationship dynamics through education in problem-solving and communication skills (17).

The intervention was delivered in a community setting in KZN to couples who had been in a relationship for at least six months. Marriage rates are low in this region, and many couples in established partnerships are not cohabiting (18).

Table 1 provides a summary of the content of each session. The full intervention development process is described elsewhere (16).

<insert Table 1 here>

For peer review only - http://bmjopen.bmj.com/site/about/guidelines.xhtml

2.3 Optimisation study participants

Couples were purposively sampled according to their engagement; defined in terms of how many counselling sessions the couple attended, and whether or not they engaged in CHTC as follows:

a. Attended CHTC after attending the first couples counselling session
b. Attended CHTC after attending 2-4 couples counselling sessions
c. Attended 4 couples counselling sessions but did not attend CHTC
d. Attended <4 couples counselling sessions and did not attend CHTC
e. Did not attend any couples counselling sessions, may or may not have attended CHTC

Couples in Group A were anticipated to have the strongest motivation to test together, based on their decision to get tested after only one counselling session, while those in Group B appeared to need more support to reach the decision to test together. Those in Group C engaged well with the intervention but chose not to test together, while those in Group D showed lower engagement with the intervention and decided not to get tested. Group E did not attend any couples counselling sessions, suggesting low motivation. This enabled us to explore diverse perceptions of the intervention and consider why couples chose to test or not to test.

In addition, five staff members who delivered the intervention were interviewed.

2.4 Recruitment procedures

Recruitment was conducted indirectly by community members who had acted as community advisory board members during the Uthando Lwethu trial. When couples were still in the local area, the community member gave each partner a card inviting them to contact the research team if they were interested in discussing their experiences of the intervention.

The staff members were invited to interview opportunistically based on availability.

For peer review only - http://bmjopen.bmj.com/site/about/guidelines.xhtml

2.5 Interview procedures

Semi-structured qualitative interviews were conducted face-to-face in the community by a gender-matched qualitative researcher (TM; female, MSc or LM; male, MSc). TM and LM are research assistants from the local community trained in qualitative interviewing skills by KM. KM is a female health psychologist based in the UK. TM, LM and KM were not involved in the original intervention study. Partners were interviewed separately, and where possible, simultaneously. The interviews took place from August-October 2019, and informed consent was taken at the start of each interview.

Participants' experiences of the two group sessions were explored at the start of the interview. Questions were then tailored according to whether the couple had attended counselling and/or CHTC, to explore their experiences of these events or to ask how they decided not to attend (Supplementary File 2). Participants were reimbursed 120 ZAR ($8). Interviews were audio-recorded, and researchers made field notes after each interview which were discussed with the team.

The interviews were conducted in isiZulu and translated into English during transcription either by the researcher or a research assistant. TM and LM read the English translation of each interview to ensure they were happy this was an accurate record. Researchers completed a debrief form after each interview to encourage reflection.

The staff interviews were conducted in English and transcribed verbatim. One interview was conducted in person (LM), while the other four were conducted from the UK by video call (KM; female, PhD, employed as a health psychology researcher). Informed consent was taken in advance on-site. The staff interview schedule explored experiences of delivering the intervention (Supplementary File 3).

2.6 Patient and Public Involvement

For peer review only - http://bmjopen.bmj.com/site/about/guidelines.xhtml

During an in-depth half-day workshop, a community working group of eight volunteers from the local community contributed to the interpretation of interview findings and decisions about optimising the intervention. The group included one couple who had taken part in the Uthando Lwethu intervention, one couple who were part of the community working group during the trial, and four individuals who were new to the intervention.

In addition, a participant feedback event was organised in which findings from the qualitative interviews were presented back to couples and staff members who had participated in interviews, and a discussion held about their perceptions of the findings.

For peer review only

2.7 Analysis

The interviews were analysed by KM using the Table of Changes from the PBA (19). The Table of Changes provides a technique for rapid qualitative analysis involving the collation of positive and negative quotes about each aspect of the intervention, in order to identify underlying beliefs that could influence engagement with the intervention and target behaviours (i.e. CHTC). Using these quotes, KM identified possible optimisations to the intervention to address barriers and promote facilitators to CHTC, drawing on theory, evidence, and couples testing guidance (1). These possible optimisations were discussed in detail with an expert stakeholder group, including all co-authors, Lynae Darbes (health psychologist and couples counsellor) and the community working group. The optimisations were prioritised using the MoSCoW framework (Must have, Should have, Could have, Would like if time permits) in order to identify those that were essential to promote CHTC (20). This pragmatic, rapid qualitative analysis approach was consistent with the aim of the study to identify optimisations to the intervention through understanding barriers to the target behaviour and experiences of the intervention.

For peer review only - http://bmjopen.bmj.com/site/about/guidelines.xhtml

Guiding principles were developed based on the detailed understanding of barriers to CHTC built up during the qualitative interviews, along with the team's expertise in evidence and theory relating to CHTC. This was an iterative process that aimed to identify key objectives for the intervention to achieve in this context, alongside key features that could be used to achieve these objectives.

A logic model was developed to show how the optimised intervention components were anticipated to influence CHTC uptake. The optimised intervention components were mapped on to constructs from the Theoretical Domains Framework (TDF) (Cane et al. 2012) and Capability Opportunity Motivation-Behaviour (COM-B) model (Michie et al. 2011). The TDF was chosen as this identified 14 domains from across a wide range of behaviour change theories and uses common terminology to describe approaches to behaviour change.

3. Results

A sub-sample of the 168 couples who took part in the Uthando Lwethu intervention were invited to qualitative interviews (n=36 couples). Thirty-two couples agreed to participate, of whom 20 couples were interviewed. In addition, one couple agreed to participate, but only the female partner was interviewed as the interview with the male partner could not be scheduled. Therefore, in total forty-one participants (aged 25-59 years) took part in an individual interview, with four couples interviewed from each of the five target groups. The mean interview duration was 57 minutes.

The staff members interviewed included the study manager and four staff members who delivered group sessions and couples counselling.

3.1 How can a couples-focused intervention be optimised to overcome barriers to engaging in CHTC in KwaZulu-Natal?

Table 2 shows an excerpt from the Table of Changes analysis.

For peer review only - http://bmjopen.bmj.com/site/about/guidelines.xhtml

<insert Table 2 here>

The following optimisations were identified by the stakeholder group based on the Table of Changes.

3.1.1 Individual fears about receiving an HIV positive diagnosis need to be addressed

Fear about how test results will impact on their life was a barrier to CHTC for some participants. One man who did not test during Uthando Lwethu described his fear of getting tested

"you will never not be scared when testing. You have that fear that you might be HIV positive" (C4900, Male aged 20-30, Group D).

Another participant who did not test described feeling overwhelmed by the reliance on medication that would be implied by a potentially positive result.

Interviewer: 'What are your concerns about HIV?'

Participant: 'I think it's the fact that once you become HIV positive you must know that you will be taking medication for the rest of your life. Yes, you can live long but knowing that you will be taking medication for the rest of your life is what concerns me'. (C3061, Female aged 20-30, Group E).

Based on these barriers, the stakeholder group agreed that the intervention needed to increase confidence that treatment is effective and accessible, and that being HIV positive does not have to mean sickness or losing your status and respect within the community. The group proposed that this education could be delivered by HIV positive peer mentors discussing how treatment helps keep them healthy, as having a role model was theorised to be more effective than information only.

3.1.2 Couples counselling sessions need to give couples a safe space to discuss perceived barriers to CHTC

The interviews with couples counsellors suggested that their investment in positive outcomes for the couple could lead them to provide advice and try to persuade couples to engage in CHTC.

For peer review only - http://bmjopen.bmj.com/site/about/guidelines.xhtml

‘*you speak and speak and speak and encouraging them about honesty and trust and everything but still tomorrow, no, I’m not ready to do the testing..... I kept on encouraging them that most of the people are testing negative, just to make them think about it*’ (Staff member 1, counsellor and facilitator)

Some participants also seemed to expect that the counselling session would involve advice: ‘*To get a chance with a third person who is going to advise you on how things should happen*’. (C680, Male aged 30-40, Group B)

The stakeholder group proposed that training facilitators in the value of open questioning could help enable a couple to discuss why they are not ready to test together and to come up with their own solutions. This training would encourage facilitators that it is not their responsibility to ensure couples engage in CHTC, and that their success as facilitators is not evaluated based on CHTC uptake. The Healthy Conversations Skills Training package (21) was identified as an appropriate tool to deliver this message during facilitator training. This emphasises to facilitators that their role is to help people recognise their own barriers and to ask open discovery questions to facilitate them in identifying solutions.

3.1.3 Couples need to feel ready to have conversations about CHTC outside the intervention setting

Couples in the Uthando Lwethu intervention were encouraged to discuss HIV testing with one another outside the intervention setting. However, a common barrier was that discussing HIV testing outside the supportive environment of the intervention was difficult due to the implied lack of trust.

‘*Yes, we would talk about it [testing]. He said that it means that I do not trust him, why else would I talk about this thing. You would see from the way he responds that you should not go any further with it.*’ (C0011, female aged 20-30 years, Group D)

For peer review only - http://bmjopen.bmj.com/site/about/guidelines.xhtml

This contrasts with a quote from a participant who tested with his partner after only one counselling session, describing their rationale for testing together:

'*If she is going to come back already tested while I have not tested, there might be finger-pointing, but if we find out at the same time, we might get the same shock and be able to comfort each other, and then look for a way forward*'. (C4510, male aged 40-50 years, Group A)

Concerns about trust and blame were reflected in the staff interviews too.

'*I think there's a lot of barriers when in a couple there's STI because there's a lot of blame. There's a lot of feeling that someone is having an affair so people they don't…I think they won't be comfortable*' (Staff member 4, counsellor and facilitator)

The stakeholder group identified several optimisations to the intervention which could facilitate couples to feel ready to discuss CHTC without blame. These included education about serodiscordance to overcome perceptions that infection automatically indicates infidelity, and a case study of a serodiscordant couple was identified as an effective means to convey this message. In addition, inviting peer mentors from the local community to single-gender sessions to facilitate a dialogue about barriers to couples testing was agreed to provide information from a credible and relatable source.

As well as optimising the content of the intervention, the group also proposed optimising the structure to facilitate couples to feel ready to test together. The original intervention involved one single-gender group session, which was highly valued by men and women as a chance to talk openly about relationship issues.

'*Do you know how difficult it is to talk when your boyfriend is here? You cannot be open to talk about whatever, but when you are with other women you can talk about whatever concerning him*' (C0011, Female aged 20-30 years, Group D)

For peer review only - http://bmjopen.bmj.com/site/about/guidelines.xhtml

Staff members also noticed that participants were more willing to have open discussions during the single-gender group session. Therefore, the stakeholder group agreed that the total time for participants in single-gender sessions could be increased in order to facilitate an open dialogue around barriers to HIV testing and associated implications at an earlier point in the intervention. This was theorised to help couples feel more ready to discuss testing together outside the intervention.

3.2 What are the theorised mechanisms of action through which the optimised intervention can change behaviour?

In order to inform the process of optimising the intervention, guiding principles were developed based on these findings regarding the main barriers to CHTC, see Table 3. These emphasise that the intervention needs to increase intimacy between couples, encourage effective communication about CHTC, and increase understanding.

<Insert Table 3 here>

A logic model was developed to show how this optimised intervention is theorised to promote CHTC uptake (Figure 2). As in Uthando Lwethu, transforming motivations remains a key mechanism through which the intervention seeks to increase CHTC uptake. This construct comes from communal coping theory (Lewis et al. 2006), which proposes that by improving relationship functioning and communication within a couple, motivation for looking after health can be shifted from the individual to the couple. In Uthando Lwethu, this approach informed the couples counselling sessions which aimed to improve relationship functioning and communication skills, thereby facilitating difficult discussions that partners may wish to have and indirectly influencing CHTC uptake.

The optimised intervention still seeks to promote transformation of motivation amongst couples, but alongside this it has a more direct focus on increasing understanding of the consequences of

For peer review only - http://bmjopen.bmj.com/site/about/guidelines.xhtml

testing, changing outcome expectancies, and managing negative emotions around HIV testing. This was informed by the qualitative interviews which suggested some people needed further support to feel ready to test for HIV. Therefore, the optimised intervention is theorised to operate both through an indirect pathway of improving relationship functioning and intimacy, but also through a more direct pathway of overcoming barriers to CHTC. Key differences from Uthando Lwethu are shown in red.

<*Insert Figure 2 here*>

4. Discussion

This study adopted the person-based approach to explore couples' and facilitators' experiences and perceptions of an intervention to promote CHTC uptake in KZN, South Africa.

Suggested optimisations to promote CHTC included group discussion facilitated by peer mentors to help improve outcome expectancies about living with HIV and address concerns about the impact on relationships, a more explicit focus on discussing barriers to CHTC during couples' counselling sessions, and more time in single-gender sessions in order to facilitate open discussion about HIV.

The use of rigorous qualitative methods was a strength of this study as it provided in-depth understanding of facilitators and barriers to couples' HIV testing, which informed optimisation of the intervention components. This is in line with recommendations that qualitative research be used to explore how HIV interventions might change behaviour to help enhance effectiveness (22). A significant limitation was that the interviews were conducted approximately five years after the intervention was delivered. Whilst qualitative interviews always rely on the reconstruction of experiences through the participant's narrative lens (23), the significant time gap meant that the experiences shared with us were particularly subject to the influence of subsequent events and the

For peer review only - http://bmjopen.bmj.com/site/about/guidelines.xhtml

For peer review only

passing of years, and should be interpreted carefully. In an ongoing trial of the optimised intervention, process interviews will be conducted to assist in understanding barriers and facilitators to CHTC at the time couples are making this decision.

A challenge in conducting the qualitative interviews was that while participants openly discussed perceived barriers to CHTC, very few responded to questions about aspects of the intervention they disliked, preferring to answer that they were very happy with everything. The use of vignettes about a fictional person may have helped participants feel comfortable to provide more open feedback.

Our finding that couples can find it difficult to discuss CHTC without connotations of blame is consistent with qualitative research in Sub-Saharan Africa showing that people overestimate the likelihood of becoming infected after a one-off sexual encounter and are therefore reluctant to test due to concerns that the test will reveal they have been unfaithful (13).The perception that discordancy is related to infidelity has been theorised to be exacerbated by social norms for multiple partners; low marriage rates; campaigns that associate HIV prevention with monogamy; and lack of knowledge about the potential for long-term differences in HIV status within couples represented by serodiscordance (12).

Misunderstandings about serodiscordance and the associated blame presents a significant challenge to implementing CHTC, and supports the need for a supportive environment for couples to discuss HIV testing, with a focus on ensuring the best future together rather than considering past infidelities, as well as education about serodiscordance. This provided a strong rationale for the theoretical shift in the logic model towards addressing perceived barriers more directly, by increasing knowledge of HIV infection and possible outcomes of testing, and exploring negative emotions around CHTC. This is in line with Protection Motivation Theory (24), in that the optimised intervention seeks to influence couples' threat perceptions and increase their self-efficacy for coping with the threat. However, unlike many interventions which aim to raise perceived risk to change behaviour, this intervention seeks to reduce the perceived risk that CHTC will threaten a couple's

For peer review only - http://bmjopen.bmj.com/site/about/guidelines.xhtml

trust. As well as being grounded in theory, the optimisations proposed by this study are evidence-based as effective strategies for facilitating CHTC uptake, including understanding the benefits of CHTC, and inviting peer mentors to facilitate discussions (25, 26).

The study offered novel insights into the experiences of lay counsellors from the community delivering a couples-focused intervention for HIV testing. As asking open questions is known to be effective to support behaviour change, whilst telling or giving advice can lead to disagreement from the recipient (27), the findings from this study suggested that additional training for counsellors to support open questioning and absolve personal responsibility for couples' decisions may be beneficial.

The optimised intervention will be evaluated in a study in KZN, and CHTC uptake will be compared with uptake in the intervention arm of the original RCT. The process of optimisation could be further streamlined in future research by adopting a flexible, adaptive trial design that allows modifications to be made in line with ongoing qualitative process research, such that barriers can be addressed as they come to light (28).

5. Conclusions

In-depth qualitative interviews identified key barriers to engaging with CHTC, including concerns about trust in the relationship, and a lack of open discussion about barriers to CHTC during counselling sessions. This informed proposed optimisations to an existing couples-focused intervention in line with evidence and theory to overcome these barriers, including the addition of a discussion with peer mentors, education about serodiscordance, and training facilitators to use open questions to explore couples' barriers to CHTC. The use of the person-based approach to intervention optimisation ensured that these changes were grounded in participants' experiences and unique psychosocial context.

For peer review only - http://bmjopen.bmj.com/site/about/guidelines.xhtml

Acknowledgements:

Thank you to all participants in this research study.

The following people have provided helpful comments or suggestions, or have otherwise enabled this work: Dr Tawanda Makusha (HSRC), and Prof Lynae Darbes (University of Michigan).

Funding:

This report is independent research supported by the National Institute for Health Research using Official Development Assistance (ODA) funding (NIHR Global Health Research Professorship, Professor Nuala McGrath, RP-2017-08-ST2-008). KM, TM, LM, NN, ZF and NMcG were supported by this funding. The views expressed in this publication are those of the author(s) and not necessarily those of the NHS, the National Institute for Health Research or the Department of Health and Social Care.

Competing interests: The authors have no competing interests to declare.

Authors' contributions: LM and TM conducted the interviews. KM trained the interviewers, conducted some staff interviews, analysed the data, and wrote the manuscript. AVH, TN, PJ, VH, HVR and NM contributed to the original intervention design and conducted the 'Uthando Lwethu' trial, as well as planning and organising the present qualitative study. NN coordinated the participant recruitment, data collection and community working group and participant feedback events. ZF contributed to intervention optimisation. All authors contributed to decisions about intervention optimisations and reviewed the manuscript.

Data sharing statement: The interview transcripts will not be made available to protect participants' anonymity.

For peer review only - http://bmjopen.bmj.com/site/about/guidelines.xhtml

References

1. World Health Organization. Guidance on couples HIV testing and counselling including antiretroviral therapy for treatment and prevention in serodiscordant couples: recommendations for a public health approach. 2012.
2. National Department of Health. National HIV Testing Services: Policy. 2016.
3. Kilembe W, Wall KM, Mokgoro M, Mwaanga A, Dissen E, Kamusoko M, et al. Implementation of couples' voluntary HIV counseling and testing services in Durban, South Africa. BMC Public Health. 2015;15(1):601.
4. Wall K, Allen S. Incentives to improve couples' HIV testing uptake and cost-effectiveness. The Lancet Global Health. 2017;5(9):e847-e8.
5. Vandormael A, Cuadros D, Kim HY, Barnighausen T, Tanser F. The state of the HIV epidemic in rural KwaZulu-Natal, South Africa: a novel application of disease metrics to assess trajectories and highlight areas for intervention. Int J Epidemiol. 2020.
6. Kim HY, Harling G, Vandormael A, Tomita A, Cuadros DF, Barnighausen T, et al. HIV seroconcordance among heterosexual couples in rural KwaZulu-Natal, South Africa: a population-based analysis. J Int AIDS Soc. 2020;23(1):e25432.
7. Matthews LT, Moore L, Crankshaw TL, Milford C, Mosery FN, Greener R, et al. South Africans with recent pregnancy rarely know partner's HIV serostatus: implications for serodiscordant couples interventions. BMC Public Health. 2014;14(1):843.
8. World Health Organization. Guideline on when to start antiretroviral therapy and on pre-exposure prophylaxis for HIV. 2015.
9. United Nations General Assembly. United Nations General Assembly. Political Declaration on HIV and AIDS: on the Fast-Track to accelerating the fight against HIV and to ending the AIDS epidemic by 2030. . New York, USA2016.
10. Darbes LA, McGrath NM, Hosegood V, Johnson MO, Fritz K, Ngubane T, et al. Results of a couples-based randomized controlled trial aimed to increase testing for HIV. JAIDS Journal of Acquired Immune Deficiency Syndromes. 2019;80(4):404-13.
11. Yardley L, Morrison L, Bradbury K, Muller I. The person-based approach to intervention development: application to digital health-related behavior change interventions. J Med Internet Res. 2015;17(1):e30.
12. Parker L, Pettifor A, Maman S, Sibeko J, MacPhail C. Concerns about partner infidelity are a barrier to adoption of HIV-prevention strategies among young South African couples. Cult Health Sex. 2014;16(7):792-805.
13. Conroy AA. 'It means there is doubt in the house': perceptions and experiences of HIV testing in rural Malawi. Culture, Health & Sexuality. 2014;16(4):397-411.
14. Pettifor A, MacPhail C, Nguyen N, Rosenberg M, Parker L, Sibeko J. Feasibility and acceptability of Project Connect: a couples-based HIV-risk reduction intervention among young couples in Johannesburg, South Africa. AIDS Care. 2014;26(4):476-82.
15. Duncan E, O'Cathain A, Rousseau N, Croot L, Sworn K, Turner KM, et al. Guidance for reporting intervention development studies in health research (GUIDED): an evidence-based consensus study. BMJ Open. 2020;10(4):e033516.
16. Darbes LA, van Rooyen H, Hosegood V, Ngubane T, Johnson MO, Fritz K, et al. Uthando Lwethu ('our love'): a protocol for a couples-based intervention to increase testing for HIV: a randomized controlled trial in rural KwaZulu-Natal, South Africa. Trials. 2014;15(1):64.
17. Markman HJ, Renick MJ, Floyd FJ, Stanley SM, Clements M. Preventing marital distress through communication and conflict management training: a 4- and 5-year follow-up. J Consult Clin Psychol. 1993;61(1):70-7.
18. Hosegood V, McGrath N, Moultrie T. Dispensing with marriage: Marital and partnership trends in rural KwaZulu-Natal, South Africa 2000-2006. Demographic Research. 2009;20:279-312.

For peer review only - http://bmjopen.bmj.com/site/about/guidelines.xhtml

19. Bradbury K, Morton K, Grist R, Band R, Van Woezik A, Connelly D, et al. Using the Person-Based Approach to optimise a digital intervention for the management of hypertension. PLoS One. 2018;13(5):e0196868.
20. Bradbury K, Watts S, Arden-Close E, Yardley L, Lewith G. Developing digital interventions: a methodological guide. Evid Based Complement Alternat Med. 2014;2014.
21. Vogel C, Lawrence W, Cradock S, Ntani G, Tinati T, Jarman M, et al. Healthy Conversation Skills: increasing competence and confidence in front-line staff. Public Health Nutr. 2012;17:1-8.
22. Camlin CS, Seeley J. Qualitative research on community experiences in large HIV research trials: what have we learned? J Int AIDS Soc. 2018;21 Suppl 7:e25173.
23. Randall W, Phoenix C. The problem with truth in qualitative interviews: reflections from a narrative perspective. Qualitative Research in Sport and Exercise. 2009;1:125-40.
24. Rogers RW. Cognitive and psychological processes in fear appeals and attitude change: A revised theory of protection motivation. Social psychophysiology: A sourcebook. 1983:153-76.
25. Nannozi V, Wobudeya E, Matsiko N, Gahagan J. Motivators of couple HIV counseling and testing (CHCT) uptake in a rural setting in Uganda. BMC Public Health. 2017;17(1):104.
26. Adeomi AA, Adeoye OA, Asekun-Olarinmoye EO, Abodunrin OL, Olugbenga-Bello AI, Sabageh AO. Evaluation of the Effectiveness of Peer Education in Improving HIV Knowledge, Attitude, and Sexual Behaviours among In-School Adolescents in Osun State, Nigeria. AIDS Res Treat. 2014;2014:131756.
27. Mason P, Butler C. Health Behavior Change: A Guide For Practitioners2010.
28. Pallmann P, Bedding AW, Choodari-Oskooei B, Dimairo M, Flight L, Hampson LV, et al. Adaptive designs in clinical trials: why use them, and how to run and report them. BMC Med. 2018;16(1):29-.

For peer review only

For peer review only - http://bmjopen.bmj.com/site/about/guidelines.xhtml

Table 1. Uthando Lwethu intervention content.

Session	Content
Group session 1	A mixed gender half-day session with approximately 15-20 couples. Information provision on TB, HIV, contraception and alcohol. This was designed as a baseline session for all couples before randomisation, and was not not part of the intervention content. Couples were randomised to the intervention or control group at the end of this session.
Group session 2	A single gender half-day session including discussions about relationship dynamics, HIV treatment, gender norms, and practical skills sessions on using condoms and learning communication techniques.
Couples counselling session 1	A 90-120 minute counselling session including discussion of relationship expectations, communication skills and goal-setting.
Couples counselling session 2	A 90-120 minute counselling session to develop communication skills, discuss barriers to achieving their goals and engage in problem-solving, and enhance positive relationship dynamics and intimacy using activities to help focus on what they like about their relationship and their partner.
Couples counselling session 3	A 90-120 minute counselling session to continue building communication and problem-solving skills.
Couples counselling session 4	A 90-120 minute counselling session to discuss maintenance of any behaviour changes, goal-setting for the future and identify possible challenges they might encounter that could impact their relationship.

For peer review only

For peer review only - http://bmjopen.bmj.com/site/about/guidelines.xhtml

Table 2. Excerpt from Table of Changes analysis.

Intervention Component	Participant	Positive comments	Negative comments	Possible Change	Reason for Change IMP; EAS; REP; EXP; NCON; NC	MoSCoW MUST do SHOULD do COULD do WOULD like
Single gender session	4511, Female aged 30-40, group A	It would be girls alone and boys by themselves and you would talk about the problems that you come across with your partner. And you would get solutions for if the problem occurs again, this is how you could face it. Interviewer: Oh. So, how was it? Participant: It was fun because we were comfortable, you could talk comfortably. There was no one with a secret.		Increase the amount of time spent in single gender sessions to allow more opportunity for open discussion amongst peers and help couples feel more ready for testing.	IMP, REP	Should have
CHTC	3960, Male aged 30-40, group D		I think if you check simultaneously with your partner, if there come different results, its better different results come out if you are not testing together and you will have to answer why results are like that and how to solve such a problem if it arises	Fear of serodiscordance needs to be addressed, perhaps through peer mentors, education about serodiscordance.	IMP	Must

For peer review only - http://bmjopen.bmj.com/site/about/guidelines.xhtml

Table 3. Guiding principles to inform the optimisation of the intervention

**Design objectives**	**Key intervention features**
Help couples feel close to each other, recognising the positive aspects of their relationship and providing motivation to look after their health together.	- Activities to promote focus on the positive aspects of the relationship.
Encourage effective communication to address fears and outcome expectancies about testing.<br><br>Address negative emotions.	- Ask open questions to enable couples to identify their barriers to CHTC and come up with their own solutions.<br>- Provide effective communication skills training to help couples explore their perceived barriers to testing together.
Increase understanding about risk of HIV transmission, serodiscordance, and effectiveness of treatment.	- Interactive single-gender group activities<br>- Open dialogue facilitated by a peer mentor with experience of couples HIV testing<br>- Provide standard guidance on HIV prevention to reduce risk of transmission, e.g. condom demonstration.

For peer review only - http://bmjopen.bmj.com/site/about/guidelines.xhtml

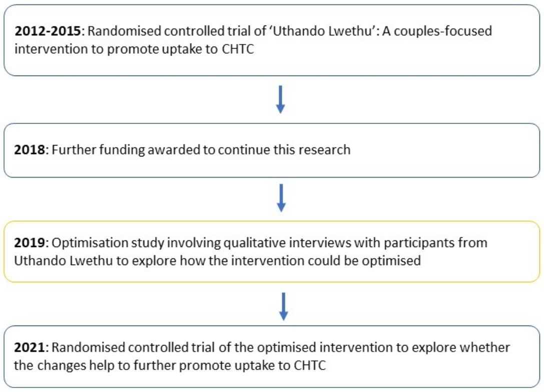

Figure 1. Stages of research leading up to and from present optimisation study.

338x190mm (96 x 96 DPI)

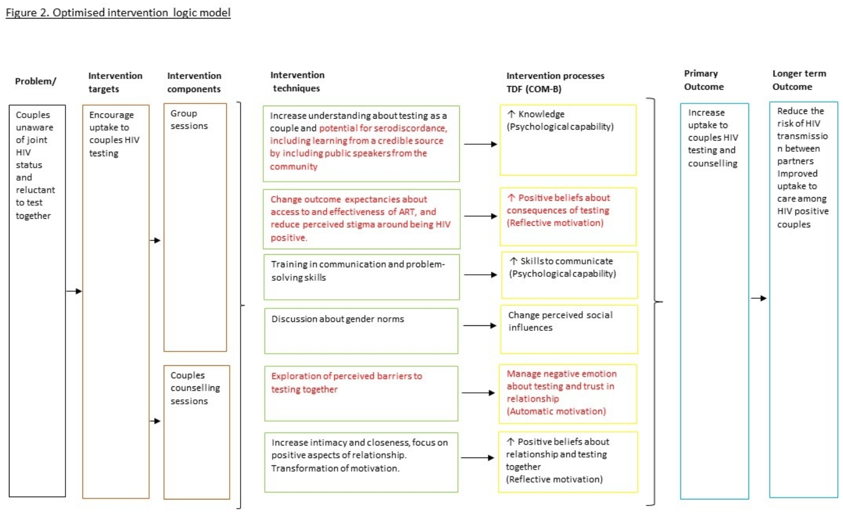

Figure 2. Optimised intervention logic model.

338x190mm (96 x 96 DPI)

Consolidated criteria for reporting qualitative studies (COREQ): 32-item checklist

Developed from:
Tong A, Sainsbury P, Craig J. Consolidated criteria for reporting qualitative research (COREQ): a 32-item checklist for interviews and focus groups. *International Journal for Quality in Health Care*. 2007. Volume 19, Number 6: pp. 349 – 357

YOU MUST PROVIDE A RESPONSE FOR ALL ITEMS. ENTER N/A IF NOT APPLICABLE

No. Item	Guide questions/description	Reported on Page #
**Domain 1: Research team and reflexivity**		
Personal Characteristics		
1. Interviewer/facilitator	Which author/s conducted the interview or focus group?	Page 9 and 10
2. Credentials	What were the researcher's credentials? E.g. PhD, MD	Page 9 and 10
3. Occupation	What was their occupation at the time of the study?	Page 9 and 10
4. Gender	Was the researcher male or female?	Page 9 and 10
5. Experience and training	What experience or training did the researcher have?	Page 9
Relationship with participants		
6. Relationship established	Was a relationship established prior to study commencement?	8
7. Participant knowledge of the interviewer	What did the participants know about the researcher? e.g. personal goals, reasons for doing the research	Supplementary Files 2 and 3
8. Interviewer characteristics	What characteristics were reported about the interviewer/facilitator? e.g. Bias, assumptions, reasons and interests in the research topic	Supplementary Files 2 and 3
**Domain 2: study design**		
Theoretical framework		
9. Methodological orientation and Theory	What methodological orientation was stated to underpin the study? e.g. grounded theory, discourse analysis, ethnography, phenomenology, content analysis	Page 7 and 10
Participant selection		
10. Sampling	How were participants selected? e.g. purposive, convenience, consecutive, snowball	Page 8
11. Method of approach	How were participants approached? e.g. face-to-face, telephone, mail, email	Page 9

For peer review only

12. Sample size	How many participants were in the study?	Page 11
13. Non-participation	How many people refused to participate or dropped out? Reasons?	Page 9
Setting		
14. Setting of data collection	Where was the data collected? e.g. home, clinic, workplace	Page 9
15. Presence of non-participants	Was anyone else present besides the participants and researchers?	N/A
16. Description of sample	What are the important characteristics of the sample? e.g. demographic data, date	Page 11
Data collection		
17. Interview guide	Were questions, prompts, guides provided by the authors? Was it pilot tested?	Supplementary Files 2 and 3
18. Repeat interviews	Were repeat inter views carried out? If yes, how many?	N/A
19. Audio/visual recording	Did the research use audio or visual recording to collect the data?	Page 9
20. Field notes	Were field notes made during and/or after the inter view or focus group?	Page 9
21. Duration	What was the duration of the inter views or focus group?	Page 11
22. Data saturation	Was data saturation discussed?	N/A
23. Transcripts returned	Were transcripts returned to participants for comment and/or correction?	N/A
**Domain 3: analysis and findings**		
Data analysis		
24. Number of data coders	How many data coders coded the data?	Page 10
25. Description of the coding tree	Did authors provide a description of the coding tree?	N/A
26. Derivation of themes	Were themes identified in advance or derived from the data?	N/A
27. Software	What software, if applicable, was used to manage the data?	N/A
28. Participant checking	Did participants provide feedback on the findings?	Page 10
Reporting		
29. Quotations presented	Were participant quotations presented to illustrate the themes/findings? Was each quotation identified? e.g. participant number	Page 12-14
30. Data and findings consistent	Was there consistency between the data presented and the findings?	Yes
31. Clarity of major themes	Were major themes clearly presented in the findings?	Yes
32. Clarity of minor themes	Is there a description of diverse cases or discussion of minor themes?	Yes

For peer review only

Once you have completed this checklist, please save a copy and upload it as part of your submission. When requested to do so as part of the upload process, please select the file type: *Checklist*. You will NOT be able to proceed with submission unless the checklist has been uploaded. Please DO NOT include this checklist as part of the main manuscript document. It must be uploaded as a separate file.

For peer review only

Supplementary File 2: Semi-structured interview schedule for participants in the couples-focused intervention

A. Introduction and thanks
My name is ************ and I am working for the 'Egugu Lethu project' which is being conducted by the Human Sciences Research Council and the University of Southampton, UK.<br><br>First, let me thank you for agreeing to speak to me today.
**B. Project explanations**
The overall aim of the 'Egugu Lethu project is to improve the couples counselling intervention that we used in the Uthando Lwethu study and to conduct a new study to test the improved intervention success in promoting couples HIV testing and counselling.<br><br>This interview is part of the first steps towards improving the previously tested intervention. We are conducting interviews with individual members of couples from the Uthando Lwethu study to explore their experiences of the intervention and it's role, if any, in contributing to their decision regarding uptake of couples HIV testing and counselling.

Interview explanations

Today I would like to ask you about your experiences of participating in the Uthando Lwethu Study. We would really like to hear your experiences, opinions and observations. I will ask you a series of questions from my guideline, but please feel free to add in extra comments that you think are important.
[I will be taping / I will not be taping] this interview and taking a few notes to aid my memory as we go along but I will not write down your name or the name of your partner.
I will not be using a questionnaire but have a list of topics that I'd like us to try and cover today. Think of our session as a conversation and that you can ask me questions too.

Great, so now we have completed the formalities, is it fine with you that we start?

What is your name?

And the name of your partner who participated with you in Uthando Lwethu Study?

General experience of the Uthando Lwethu Study

Before we start talking in detail about your experiences of specific parts of the Uthando Lwethu Study, can you tell me about the first day when you were approached about the study? What was that like for you?
Can you remember why you decided to sign up for the study?

For peer review only - http://bmjopen.bmj.com/site/about/guidelines.xhtml

I'm really interested in hearing about your experiences of the Uthando Lwethu study, Can you tell me what you remember about being in the study?

How did you find the sessions?

Does anything stand out in your memory about the study? What do you remember and why?

Can you tell me about what difference Uthando Lwethu made for you, if any?

Experience of the first group session

As a quick reminder, the first group session was where we gave some information about health to the group of couples attending together and at the end we had the lottery where you learned you and your partner were going to receive the Uthando Lwethu couples counselling sessions .

Can you tell me all about what the first group session was like for you?

[Note to interviewer: information given was on TB, reproductive health/HIV), and a list of community resources for health care and treatment was given]

What information did you find most helpful? And why?

What other information would you have liked?

How was it to see other couples from your area at the same session? Did you know anyone else there? Who?

Can you remember how you sat in the first session? Did you sit with your partner, or with other men/women?

How did you interact with other couples from your area?

Can you tell us about anything you remember learning in the first session?

Experience of the second group session

The second group session was where you met in [men-only / female only] groups and discussed roles, violence and power within relationships. There was also skills training for communication and risk reduction. What was your experience of that second group session?

What was most helpful in this session? Why?

What other information would you have liked? Why?

For peer review only - http://bmjopen.bmj.com/site/about/guidelines.xhtml

Can you tell me about any conversations you had with your partner about that session afterwards?

What did you discuss? Once off or repeatedly?

Were there any particular topics that you discussed in particular? [prompt for specific exercises / content that was included in the session to help participants remember]

Was one same gender group session enough or would you have liked more? Why?

Couples sessions

The next section is dependent on whether they attended the couples' sessions. Only one block of questions from this section is applicable in any particular individual interview.

a. For those who did NOT attend couples sessions at all

If you remember, you were then offered 4 couples' sessions *with* <counsellor's name> Can you tell me about why you did not attend the couples' sessions that were on offer?

How did you reach that decision?

Was this your choice or your partner's, or mutual?

What were your main concerns about the couples' sessions?

Could the study have done something differently that might have changed your mind? Or your partner's mind? Or made it possible logistically?

b. For those who attended one or more couples sessions

What was your main reason for deciding to attend the couples' sessions?

How did you reach that decision?

Did you have any concerns or worries about attending?

Do you remember your counsellor? [check they mean the same person recorded on page 1]. I am interested in how you felt about the couples' sessions that you attended.

If I asked you to describe ~~them~~ *the sessions in one or two sentences*, what would you say?

For peer review only - http://bmjopen.bmj.com/site/about/guidelines.xhtml

*Did you feel you were able to communicate your point of view and thoughts to your **partner** in the counselling session? And did your partner? Can you tell me about why?*

*Did you feel you were able to communicate your point of view and thoughts to the **counselor** in the counselling session? Can you tell me about why?*

How do you think your partner felt about it?

Was there anything you would change?

***[If they used all 4 sessions]:** Was the number of sessions enough*

***[If they used less than 4 sessions]:** Can you tell me about why you stopped attending when you did?*

Would having an additional session made a difference? Was another session likely to have changed your decision about testing?

Can you tell us about anything you remember learning from the sessions? [prompt for specific tasks or exercises / content to help participants remember]

Did you discuss anything about the session or from a session with your partner afterwards? What did you talk about?

Do you remember some of the homework exercises that the counsellor gave you? How did you and your partner get on with the homework?

(If still together) Can you tell me how you feel about communicating with your partner now?

Are you still practicing the skills taught? Why or why not? If not, how long after did you stop using the skills?

Cohabitation

The next section is dependent on the couple cohabitation status at the time of their participation in the Uthando Lwethu study. <u>Only one block</u> of questions from this section is applicable in any particular individual interview.

a. For those cohabiting at the time

At the time of the Uthando Lwethu study, you were living with your partner.

Can you tell me about what it was like doing the Uthando Lwethu study while you were living at home with your partner?

Did you want to talk to your partner about Uthando Lwethu?

If yes – did you have the opportunity to talk to them at home?

If no – why not?

Was anything different at home when you were both doing Uthando Lwethu?

For peer review only - http://bmjopen.bmj.com/site/about/guidelines.xhtml

b. For those NOT cohabiting at the time

At the time of the Uthando Lwethu study, you were not living with your partner.

Can you tell me about what it was like doing the Uthando Lwethu study while you were living separately from your partner?

Did you want to talk to your partner about Uthando Lwethu?

If yes – did you have the opportunity to talk to them? Where did the conversations happen?

If no – why not?

Was anything different when you met up while you were both doing Uthando Lwethu?

HIV testing

The next section is for ALL individuals

What do you think about HIV testing?

And testing with a partner together?

What are your main concerns about HIV testing?

How could we address those concerns?

The next section is dependent on whether the couple tested for HIV as a couple during the Uthando Lwethu study. Only one block of questions from this section is applicable in any particular individual interview.

a. For those who did not test for HIV as a couple during the Uthando Lwethu Study

I'm really interested in your decision around whether to test for HIV with your partner. It looks like you decided not to test together during the study, can you tell me more about that decision?

Did you as a couple discuss testing? Why or why not? How did you reach a decision?
How did you feel about getting tested?

How did your partner feel?

For peer review only - http://bmjopen.bmj.com/site/about/guidelines.xhtml

What were your main concerns? What could be done to address those concerns? Eg more information? sessions? support?

What do you think might have been the benefits of testing for you?

How did you resolve any conflict with your partner?

Did you test together anytime after the study had finished? How long after the study?

Do you know your partner's status? How do you know this?

Do you think the group sessions or couples counseling sessions contributed to your / your partner's decision?

b. For those who tested for HIV as a couple during the Uthando Lwethu Study

Have you ever tested together before Uthando Lwethu?
If yes - how did you decide to do this?

Can you tell me about why you decided to test together during the Uthando Lwethu study?

How did you reach a decision?

How did you feel about getting tested?

How did your partner feel?
Did you have any concerns about the testing? What could be done to address those concerns? Eg more information? sessions? support?

How did you resolve any conflict with your partner about deciding whether to get tested?

How did you arrange the testing?

How did you feel after the testing?

Did you talk about it with your partner? What do you think were the benefits of testing for you?

How would you encourage other couples to have HIV testing?

For peer review only - http://bmjopen.bmj.com/site/about/guidelines.xhtml

Do you think the group sessions or couples counseling sessions contributed to your / your partner's decision?

If yes: What part of the intervention encouraged you to be tested?

If not, why not?

For all individuals

In the new study, we may offer testing for sexually transmitted infections (STIs), specifically testing for chlamydia, syphilis, gonorrhoea and trichomoniasis. However we don't know if this would be useful for people, or what some of the concerns might be in having STI testing.

Can you tell me what you think about the idea of testing couples for sexually transmitted infections?

If we had also invited you to consider STI testing as part of the couples HIV testing session, how would you have felt about that?

Would you have taken up an offer for STI testing? Why/why not?

The STI testing would mean giving a sample that would be sent away to the labs for testing and you and your partner would come back to receive the results, For men, this would involve providing a urine sample. How would you feel about that?

For women, this would mean privately taking a vaginal swab. Many women do this themselves, or the nurse could help you with if you wanted. How would you feel about that?

Overall, what do you think about the Uthando Lwethu study for other people?

We are hoping to make some changes to Uthando Lwethu to make it even better for people. Can you think of any other health or relationship topics you would have liked to know more about in group session 1, group session 2, or the counselling sessions?

Can you think of anything that would have made Uthando Lwethu better for you?

And was there any part of Uthando Lwethu that you think was not needed or was not helpful to you?

For peer review only - http://bmjopen.bmj.com/site/about/guidelines.xhtml

Supplementary File 3. Semi-structured interview schedule for staff members.

**A. Introduction and thanks**
My name is ************ and I am working for the 'Egugu Lethu project' which is being conducted by the Human Sciences Research Council and the University of Southampton, UK.<br><br>First, let me thank you for agreeing to speak to me today.
**B. Project explanations**
The overall aim of the Egugu Lethu project is to improve the couples counselling intervention that we used in the Uthando Lwethu study and to conduct a new study to test the improved intervention success in promoting couples HIV testing and counselling.<br><br>The first step of optimising the previously tested intervention is to conduct interviews with individual members of couples from the Uthando Lwethu study and with staff members who were involved in delivering the intervention components. In staff interviews we will discuss staff experiences of the previous study and possible barriers to couples' decisions to test for HIV together.

Interview explanations

Today I would like to ask you about your experiences as an Uthando Lwethu Study staff member. We would really like to hear your experiences, opinions and observations. I will ask you a series of questions from my guideline, but please feel free to add in extra comments that you think are important. [I will be taping / I will not be taping] this interview and taking a few notes to aid my memory as we go along but I will not write down your name. I will not be using a questionnaire but have a list of topics that I'd like us to try and cover today. Think of our session as a conversation and that you can ask me questions too.

Great, so now we have completed the formalities, is it fine with you that we start?

What is your name?

What was your role in the Uthando Lwethu Study: Couples counsellor or part of the group sessions or counsellor for the HIV testing and counselling?

Can you tell me a bit about your background? I'd like to understand what your job is outside of Uthando Lwethu?

Can you tell me about the training you've had previously?

What was different in Uthando Lwethu from your usual counselling/facilitating role?

What was the same?

General experience of the Uthando Lwethu Study

For peer review only - http://bmjopen.bmj.com/site/about/guidelines.xhtml

Before we start talking in detail about your experiences and perceptions of specific parts of the Uthando Lwethu Study, I would like to hear what was it like being part of the study team overall?

How did you find the training you received before becoming a facilitator/counsellor for Uthando Lwethu?

The next section is dependent on which role the staff member had. <u>Only one block</u> of questions from this section is applicable in any particular individual interview.

For staff who attended group sessions

How many first group sessions did you attend? What was your role in the first group sessions?

what was your role in the second group sessions?

We're interested in how to improve the intervention for couples, so the next few questions are to try and find out more about how it worked last time, and what we could do better going forwards.

How did you find the first group sessions?

How did you find the second group sessions?

Did you notice any differences between the two sessions?

Can you recall what happened in a typical second group session?

What was it like following the manual for Uthando Lwethu during the second group session?

Were there any activities that stood out for you as being particularly important in the second group session?

Were there any activities that you remember being challenging for some people in the second group session?

How engaged did you feel people were? Can you tell me a story about any memorable moments you recall?

Are there any particular benefits/negatives to the single gender session?

For peer review only - http://bmjopen.bmj.com/site/about/guidelines.xhtml

How did the single gender sessions vary between groups?

What was it like to deliver this information to females/males as a man/woman yourself? Did the same / different gender dynamic work well or not really?

Could session 2 be reinforced by homework If we introduced homework at this session? do you think it would work best done as a couple of individually?

Did you feel there were any differences in the level of participation or questions between sessions?

How important do you think these group sessions were for encouraging couples to attend HIV testing together?

Do you think there was anything about the first or second group session that didn't work very well, that you think we should change for the next study? *[prompt for organizational or staff changes vs content]*

If **not** already mentioned, ask about:

In the first group session, how do you think couples found the information on:

- TB
- HIV
- HIV testing
- Contraception
- Alcohol

In the second group session, how do you think participants found the:

- Discussion around long-term relationships
- Condom demonstration
- Discussions about gender and power imbalance
- Practising communication skills

In the new study, we may offer testing for sexually transmitted infections (STIs), specifically testing for chlamydia, syphilis, gonorrhoea and trichomoniasis. Would any changes be needed in the way Group sessions 1 and 2 are conducted or their content if STI testing were offered alongside HIV testing and counselling?

How do you think couples would feel about testing for STIs together?
For men, it would involve providing a urine sample, and for women it would mean taking a vaginal swab. The samples are sent off to a lab, so the couple would need to come back to receive their results. How do you think this would work for couples in Uthando Lwethu?

For staff who conducted couples counselling sessions

For peer review only - http://bmjopen.bmj.com/site/about/guidelines.xhtml

Approximately, how many couples did you counsel?

How did you feel about the couples' counselling sessions that you led?

How do you think they were experienced by the participating couples?

Within those counselling sessions, how did you find it interacting with the female partner, as a female/male counsellor? And what about working with the male partner? Why?

Did the interaction with different couples vary? In what way did it vary and why?

Can you tell me about any reasons you can think of why some couples got more out of the counselling than others?

How do you think couples found the number of counselling sessions? Do you think having an additional session would make a difference / likely to have changed couples' decisions about testing?

How do you think the couples felt about practicing the speaker-listener technique between sessions?

Which exercises in the counselling sessions were particularly helpful for couples?

Were there any activities in the counselling sessions that you remember being challenging for some couples?

What was it like following the intervention manual in the couples counselling session?

If **not** already mentioned, ask the following:
In the couples counselling sessions, what do you think it was like for couples:

- Identifying and talking about their expectations and barriers around HIV testing
- Practising the speaker listener technique with their partner
- Planning solutions to problems together
- Setting some personal goals
- Doing the 'forgetting something good' activity, where they noticed what helpful things their partner has done for them recently
- Doing the 'words of the heart' activity where couples chose words that describe their relationship

If the participant only talks about positive examples where couples liked these things, ask:

  - *Were there any couples who struggled to do that?*

For peer review only - http://bmjopen.bmj.com/site/about/guidelines.xhtml

For peer review only

- *If so, what was that like for you?*

Why do you think some couples didn't go for HIV testing? What do you think were the barriers for them?

Can you tell me about a couple who did go for testing? Why do you think they decided to get tested?

In the new study, we may offer testing for sexually transmitted infections (STIs), specifically testing for chlamydia, syphilis, gonorrhoea and trichomoniasis. Would any changes be needed in the way the couples counselling sessions are conducted or their content if STI testing were offered alongside HIV testing and counselling?

How do you think couples would feel about testing for STIs together?
For men, it would involve providing a urine sample, and for women it would mean taking a vaginal swab. The samples are sent off to a lab, so the couple would need to come back to receive their results. How do you think this would work for couples in Uthando Lwethu?

For peer review only

For peer review only - http://bmjopen.bmj.com/site/about/guidelines.xhtml

For staff who conducted couples HIV testing and counselling during the Uthando Lwethu Study

Approximately how many couples HIV testing and counselling sessions did you conduct during Uthando Lwethu Study?

How did you feel about the couples' HIV testing and counselling sessions that you conducted?

Did the couples discuss with you how they came to the decision to test for HIV together? What reason(s) did they give?

In the new study, we may offer testing for sexually transmitted infections (STIs), specifically testing for chlamydia, syphilis, gonorrhoea and trichomoniasisdo you think offering STI testing as part of the session would change couples' interest in testing for HIV together?

To what extent do you think including STI testing in the couples testing session would change this session for you as counsellor?

How do you think couples would feel about testing for STIs together?
For men, it would involve providing a urine sample, and for women it would mean taking a vaginal swab. The samples are sent off to a lab, so the couple would need to come back to receive their results. How do you think this would work for couples in Uthando Lwethu?

For all staff at the end:

Did you have any contact with couples after ***their*** *participation in the study had ended?*

What was the nature of those interactions? [Probe for friendly / casual, neighbour, asking advice, sought out staff member's skill as counsellor etc.]

For peer review only - http://bmjopen.bmj.com/site/about/guidelines.xhtml

BMJ Open

Optimising a couples-focused intervention to increase couples' HIV testing and counselling using the person-based approach: A qualitative study in Kwa-Zulu Natal, South Africa.

Journal:	*BMJ Open*
Manuscript ID	bmjopen-2020-047408.R2
Article Type:	Original research
Date Submitted by the Author:	10-Aug-2021
Complete List of Authors:	Morton, Katherine; University of Southampton, Psychology<br>Mhlakwaphalwa, Tembeka; Human Sciences Research Council<br>Msimango, Lindani; Human Sciences Research Council<br>van Heerden , Alastair ; Human Sciences Research Council; University of the Witwatersrand Faculty of Health Sciences, School of Clinical Medicine<br>Ngubane, Thulani; Human Sciences Research Council<br>Joseph, Philip; Human Sciences Research Council<br>Ngcobo, Nathi; Human Sciences Research Council<br>Feng, Z; University of Southampton, Primary Care and Population Sciences<br>Hosegood, Victoria; University of Southampton, Department of Social Statistics and Demography, Faculty of Social Sciences<br>van Rooyen, Heidi; Human Sciences Research Council; University of the Witwatersrand<br>McGrath, Nuala; University of Southampton, School of Primary Care, Population Sciences and Medical Education, Faculty of Medicine; University of Southampton, Department of Social Statistics & Demography, Faculty of Social Sciences
**Primary Subject Heading**:	Sexual health
Secondary Subject Heading:	Public health, Qualitative research, HIV/AIDS
Keywords:	HIV & AIDS < INFECTIOUS DISEASES, PUBLIC HEALTH, QUALITATIVE RESEARCH

SCHOLARONE™
Manuscripts

I, the Submitting Author has the right to grant and does grant on behalf of all authors of the Work (as defined in the below author licence), an exclusive licence and/or a non-exclusive licence for contributions from authors who are: i) UK Crown employees; ii) where BMJ has agreed a CC-BY licence shall apply, and/or iii) in accordance with the terms applicable for US Federal Government officers or employees acting as part of their official duties; on a worldwide, perpetual, irrevocable, royalty-free basis to BMJ Publishing Group Ltd ("BMJ") its licensees and where the relevant Journal is co-owned by BMJ to the co-owners of the Journal, to publish the Work in this journal and any other BMJ products and to exploit all rights, as set out in our licence.

The Submitting Author accepts and understands that any supply made under these terms is made by BMJ to the Submitting Author unless you are acting as an employee on behalf of your employer or a postgraduate student of an affiliated institution which is paying any applicable article publishing charge ("APC") for Open Access articles. Where the Submitting Author wishes to make the Work available on an Open Access basis (and intends to pay the relevant APC), the terms of reuse of such Open Access shall be governed by a Creative Commons licence – details of these licences and which Creative Commons licence will apply to this Work are set out in our licence referred to above.

Other than as permitted in any relevant BMJ Author's Self Archiving Policies, I confirm this Work has not been accepted for publication elsewhere, is not being considered for publication elsewhere and does not duplicate material already published. I confirm all authors consent to publication of this Work and authorise the granting of this licence.

For peer review only

Optimising a couples-focused intervention to increase couples' HIV testing and counselling using the person-based approach: A qualitative study in Kwa-Zulu Natal, South Africa.

Kate Morton*[1] k.s.morton@soton.ac.uk

Tembeka Mhlakwaphalwa[2] tembekam23@gmail.com

Lindani Msimango[2] LIMsimango@hsrc.ac.za

Alastair Van Heerden[2, 3] avanheerden@hsrc.ac.za

Thulani Ngubane[2] tngubane@hsrc.ac.za

Philip Joseph[2] pjoseph@hsrc.ac.za

Nathi Ngcobo[2] NNgcobo@hsrc.ac.za

Zhixin Feng[4] Z.Feng@soton.ac.uk

Victoria Hosegood[5] V.Hosegood@soton.ac.uk

Heidi Van Rooyen[2,3] hvanrooyen@hsrc.ac.za

Nuala McGrath[4,5] N.McGrath@soton.ac.uk

1. School of Psychology, University of Southampton, UK.

2. Human Sciences Research Council, Sweetwaters, KwaZulu-Natal, South Africa.

3. SAMRC-WITS Developmental Pathways for Health Research Unit, Faculty of Health Sciences, University of the Witwatersrand, South Africa .

For peer review only

4. School of Primary Care, Population Sciences and Medical Education, Faculty of Medicine, University of Southampton, UK

5. Department of Social Statistics & Demography, Faculty of Social Sciences, University of Southampton, UK

*Corresponding author: k.s.morton@soton.ac.uk; Centre for Clinical and Community Applications of Health Psychology, B44, University of Southampton, Southampton, SO17 1BJ

For peer review only

For peer review only - http://bmjopen.bmj.com/site/about/guidelines.xhtml

Abstract:

Objectives: This qualitative study explored how to optimise a couples-focused intervention to promote couples HIV testing and counselling (CHTC).

Setting: Community setting in Kwa-Zulu Natal, South Africa.

Participants: Qualitative interviews were conducted with 20 couples who had participated in a couples-focused intervention and five staff members delivering the intervention. Partners were interviewed individually by researchers of the same gender.

Intervention: A couples-focused intervention comprised of two group sessions and four couples counselling sessions was previously shown to significantly increase uptake to CHTC in Kwa-Zulu Natal, South Africa. However, more than half of couples participating in the intervention still chose not to test together during follow-up.

Analysis: The transcripts were analysed using the table of changes from the person-based approach. Proposed optimisations were discussed with a community group to ensure the intervention was as persuasive and acceptable as possible.

Results: Many couples found it challenging to discuss CHTC with their partner due to an implied lack of trust. Optimisations to the intervention were identified to increase readiness to discuss CHTC, including education about serodiscordance, discussions about CHTC by peer mentors, and open discussion of personal barriers to CHTC during couples' counselling sessions. Additional training for staff in open questioning techniques could help them feel more comfortable to explore couples' perceived barriers to CHTC, rather than advising couples to test. A logic model was developed to show anticipated mechanisms through which the optimised intervention would increase uptake to

For peer review only - http://bmjopen.bmj.com/site/about/guidelines.xhtml

CHTC, including increasing knowledge, increasing positive outcome beliefs, and managing negative emotions.

Conclusions: In-depth qualitative research informed optimisations to a couples-focused intervention for further evaluation in South Africa to encourage uptake to CHTC. Suggestions are made for optimal methods to gain open feedback on intervention experiences where participants may be reluctant to share negative views.

Key words: Couples-focused, intervention, Couples HIV Counselling and Testing, person-based approach

For peer review only

For peer review only - http://bmjopen.bmj.com/site/about/guidelines.xhtml

Strengths and Limitations of this study

- Conducting qualitative interviews with partners separately, in their own homes, and with a researcher of the same gender helped promote rapport and facilitated open reflections about their relationship.
- Interviewing staff members responsible for delivering the intervention in addition to couples participating in the intervention provided a more in-depth understanding of potential barriers to CHTC.
- The Table of Changes from the Person-Based Approach provided a rigorous method to analyse the qualitative interviews. The use of guiding principles and a logic model ensured that optimisations were grounded in the specific psychosocial context of this population and drew on theoretical constructs.
- Conducting the interviews five years after the intervention had been delivered may have limited participants' ability to accurately recall their experiences and perceptions.
- Many participants appeared reluctant to share any negative views of the intervention, and alternative methods such as the use of vignettes may have helped obtain more critical feedback about the couples-focused intervention.

For peer review only

For peer review only - http://bmjopen.bmj.com/site/about/guidelines.xhtml

1. Introduction

Couples HIV Testing and Counselling (CHTC) is recommended by the World Health Organisation (1) and is now part of South African policy guidelines (2), however, very few couples in South Africa have tested together (3, 4). This is against a background of continued high levels of HIV prevalence, particularly in KwaZulu-Natal (KZN), South Africa. In a large community-based survey of adults in KZN, 19% of men and 41% of women were living with HIV in 2017 (5). The majority of couples in stable sexual relationships reported being unaware of their partner's HIV status (57%) (6), and testing rates remain low, particularly for men (7). CHTC can contribute to reductions in HIV incidence as a compliment to the universal test and treat policy in South Africa (8) and individual focused testing campaigns (9).

In 2012-2015, a couples-focused intervention ('Uthando Lwethu'*)* was implemented in a rural community setting in uMgungundlovu, KZN. This community has high levels of migration and unemployment, and low marriages rates, so many couples in the intervention were neither married nor cohabiting (10). At the outset, nearly 40% of the sample had never had an HIV test, and most had not shared their HIV status with their current partner (10). The intervention was shown to significantly increase uptake to CHTC, with 42% of couples attending CHTC within a 9-month follow-up period compared with 12% in the control group (10). Nonetheless, over half of couples receiving the intervention chose not to test within the study period.

In order to optimise the couples-focused intervention for future implementation, we undertook further supplementary research to explore how the intervention content and delivery could be modified to support couples to feel ready for CHTC. This paper presents the findings from a qualitative study with couples and staff involved in the Randomised Controlled Trial (RCT) of the Uthando Lwethu intervention. The person-based approach (PBA) (11) was adopted, which complements evidence and theory in developing and optimising effective interventions and has

For peer review only - http://bmjopen.bmj.com/site/about/guidelines.xhtml

been used to successfully create behaviour change interventions that are feasible, persuasive, motivating and engaging (12). This approach recommends conducting in-depth research to develop an understanding of the psychosocial context and underlying beliefs in the target population that could influence engagement with behaviour change. Guiding principles are developed to describe specific behavioural barriers for a given population and context and identify particular intervention features to overcome these barriers. In addition, this intervention optimisation process was informed by recent evidence regarding facilitators to CHTC (13-15), and a consideration of the theoretical mechanisms through which the optimised intervention might impact couples' readiness to undertake CHTC.

Our research questions were:

a. How can a couples-focused intervention be optimised to overcome barriers to engaging in CHTC in KwaZulu-Natal?
b. What are the theorised mechanisms of action through which the optimised intervention is anticipated to change behaviour?

2. Methods

2.1 Optimisation study design

Figure 1 shows how this optimisation study fitted into a wider body of research, with the orange box showing the present study.

<Insert Figure 1>

This was a qualitative study using retrospective semi-structured interviews to explore possible barriers to engaging in CHTC amongst couples participating in the Uthando Lwethu intervention. Due to the logistics of research funding and contracts, the interviews were conducted approximately five years after participants took part in the intervention. We report the optimisation process using the

For peer review only - http://bmjopen.bmj.com/site/about/guidelines.xhtml

GUIDED checklist (Guidance for reporting intervention development studies in health research) in line with best practice for reporting intervention development (16). The COREQ checklist was used for reporting (Supplementary File 1). Approval was granted by the Human Sciences Research Council (REC 2/19/10/11b) and the University of Southampton (reference number: ERGO 48702) ethics committees.

2.2 Uthando Lwethu Intervention, prior to optimisation

As this study aimed to describe the optimisation of a couples-focused intervention to promote CHTC, the original content and delivery of the Uthando Lwethu intervention trialled in the 2012-2015 randomised controlled trial (17) is described here.

The intervention was delivered in a community setting in KZN to 168 couples who had been in a relationship for at least six months. Just under two thirds of participants had had an HIV test before (63% of males and 60% of females) but only 20% of these had told their partner their HIV status. No couples had mutually disclosed their HIV status to each other (10). The mean age was 26 years and 20% of the couples were married (10).

Uthando Lwethu was a couples-focused intervention comprised of two group sessions and four couples counselling sessions that aimed to increase CHTC uptake. It was developed based on interdependence theory which states that positive relationship dynamics can lead to a transformation of motivation, in which couples are motivated to engage in health behaviours due to a focus on the relationship rather than an individual perspective. It was theorised that couples would be more willing to engage in CHTC if they have greater commitment and trust in their relationship (17). The intervention was adapted from the Prevention and Relationship Enhancement Programme (PREP), which aims to improve relationship dynamics through education in problem-solving and communication skills (18).

.

For peer review only - http://bmjopen.bmj.com/site/about/guidelines.xhtml

Table 1 provides a summary of the content of each session. The full intervention development process is described elsewhere (17).

<insert Table 1 here>

Forty two percent of couples in the intervention group attended CHTC, of whom 46% were concordant HIV-negative, 30% were concordant HIV-positive, and 24% were serodiscordant (10). In 54% of the couples who took up CHTC in the intervention group, at least one partner was testing for the first time. Fifty nine percent of the participants who were diagnosed as HIV-positive were new diagnoses (10).

2.3 Optimisation study participants

Couples were purposively sampled according to their engagement; defined in terms of how many counselling sessions the couple attended, and whether or not they engaged in CHTC as follows:

a. Attended CHTC after attending the first couples counselling session
b. Attended CHTC after attending 2-4 couples counselling sessions
c. Attended 4 couples counselling sessions but did not attend CHTC
d. Attended <4 couples counselling sessions and did not attend CHTC
e. Did not attend any couples counselling sessions, may or may not have attended CHTC

We sought to sample the same number of couples across the five groups. Couples in Group A were anticipated to have the strongest motivation to test together, based on their decision to get tested after only one counselling session, while those in Group B appeared to need more support to reach the decision to test together. Those in Group C engaged well with the intervention but chose not to test together, while those in Group D showed lower engagement with the intervention and decided not to get tested. Group E did not attend any couples counselling sessions, suggesting low motivation. This enabled us to explore diverse perceptions of the intervention and consider why couples chose to test or not to test.

For peer review only - http://bmjopen.bmj.com/site/about/guidelines.xhtml

In addition, five staff members who delivered the intervention were interviewed.

2.4 Recruitment procedures

This study took place within a community research site, which facilitated ongoing follow-up of people via contacts in the community, and generally helps ensure service provision. Therefore recruitment was conducted indirectly by community members who had acted as community advisory board members during the Uthando Lwethu trial. If couples were still in the local area, the community member gave each partner a card inviting them to contact the research team if they were interested in discussing their experiences of the intervention.

The staff members were invited to interview opportunistically based on availability.

2.5 Interview procedures

Semi-structured qualitative interviews were conducted face-to-face in the community by a gender-matched qualitative researcher (TM; female, MSc or LM; male, MSc). TM and LM are research assistants from the local community trained in qualitative interviewing skills by KM. KM is a female health psychologist based in the UK. TM, LM and KM were not involved in the original intervention study. Partners were interviewed separately, and where possible, simultaneously. The interviews took place from August-October 2019, and informed consent was taken at the start of each interview.

Participants' experiences of the two group sessions were explored at the start of the interview. Questions were then tailored according to whether the couple had attended counselling and/or CHTC, to explore their experiences of these events or to ask how they decided not to attend (Supplementary File 2). Participants were reimbursed 120 ZAR ($8). Interviews were audio-recorded, and researchers made field notes after each interview which were discussed with the team.

The interviews were conducted in isiZulu and translated into English during transcription either by the researcher or a research assistant. TM and LM read the English translation of each interview to

For peer review only - http://bmjopen.bmj.com/site/about/guidelines.xhtml

ensure they were happy this was an accurate record. Researchers completed a debrief form after each interview to encourage reflection.

The staff interviews were conducted in English and transcribed verbatim. One interview was conducted in person (LM), while the other four were conducted from the UK by video call (KM; female, PhD, employed as a health psychology researcher). Informed consent was taken in advance on-site. The staff interview schedule explored experiences of delivering the intervention (Supplementary File 3).

2.6 Patient and Public Involvement

During an in-depth half-day workshop, a community working group of eight volunteers from the local community contributed to the interpretation of interview findings and decisions about optimising the intervention. The group included one couple who had taken part in the Uthando Lwethu intervention, one couple who were part of the community working group during the trial, and four individuals who were new to the intervention.

In addition, a participant feedback event was organised in which findings from the qualitative interviews were presented back to couples and staff members who had participated in interviews, and a discussion held about their perceptions of the findings.

For both workshops, we explained how the Uthando Lwethu intervention was theorised to increase uptake to CHTC, to help participants understand the rationale behind the intervention procedures. We then gave an overview of the questions asked during the interviews, to explain the context in which the data were collected. Key quotes were selected from the interviews to demonstrate each barrier to CHTC, and these were displayed on a screen for the groups to discuss and come up with possible solutions, working in smaller groups. We then discussed their ideas as a larger group, presented other possible solutions, and the group were asked to discuss their perceptions of the

For peer review only - http://bmjopen.bmj.com/site/about/guidelines.xhtml

value and feasibility of each. The workshops were not recorded but note takers were present in each group.

2.7 Analysis

The interviews were analysed by KM using the Table of Changes from the PBA (19). The Table of Changes provides a technique for rapid inductive qualitative analysis, involving the collation of positive and negative quotes about each aspect of the intervention in order to identify underlying beliefs that could influence engagement with the intervention and target behaviours (i.e. CHTC). Using these quotes, KM identified possible optimisations to the intervention to address barriers and promote facilitators to CHTC, drawing on theory, evidence, and couples testing guidance (1). These possible optimisations were discussed in detail with an expert stakeholder group, including all co-authors, Lynae Darbes (health psychologist and couples counsellor) and the community working group. The optimisations were prioritised using the MoSCoW framework (Must have, Should have, Could have, Would like if time permits) in order to identify those that were essential to promote CHTC (20). This pragmatic, rapid qualitative analysis approach was consistent with the aim of the study to identify optimisations to the intervention through understanding barriers to the target behaviour and experiences of the intervention.

Guiding principles were developed based on the detailed understanding of barriers to CHTC built up during the qualitative interviews, along with the team's expertise in evidence and theory relating to CHTC. This was an iterative process that aimed to identify key objectives for the intervention to achieve in this context, alongside key features that could be used to achieve these objectives.

A logic model was developed to show how the optimised intervention components were anticipated to influence CHTC uptake. This was a deductive process drawing on existing theory; the optimised intervention components were mapped on to constructs from the Theoretical Domains Framework

For peer review only - http://bmjopen.bmj.com/site/about/guidelines.xhtml

(TDF) (21) and Capability Opportunity Motivation-Behaviour (COM-B) model (22) . The TDF was chosen as this identified 14 domains from across a wide range of behaviour change theories and uses common terminology to describe approaches to behaviour change.

3. Results

A sub-sample of the 168 couples who took part in the Uthando Lwethu intervention were invited to qualitative interviews (n=36 couples). Thirty-two couples agreed to participate, of whom 20 couples were interviewed. In addition, one couple agreed to participate, but only the female partner was interviewed as the interview with the male partner could not be scheduled. Therefore, in total forty-one participants (aged 25-59 years) took part in an individual interview, with four couples interviewed from each of the five target groups. The mean interview duration was 57 minutes.

The staff members interviewed included the study manager and four staff members who delivered group sessions and couples counselling.

3.1 How can a couples-focused intervention be optimised to overcome barriers to engaging in CHTC in KwaZulu-Natal?

Table 2 shows an excerpt from the Table of Changes analysis.

<insert Table 2 here>

The following optimisations were identified by the stakeholder group based on the Table of Changes.

3.1.1 Individual fears about receiving an HIV positive diagnosis need to be addressed

For peer review only - http://bmjopen.bmj.com/site/about/guidelines.xhtml

Fear about how test results will impact on their life was a barrier to CHTC for some participants. One man who did not test during Uthando Lwethu described his fear of getting tested

"you will never not be scared when testing. You have that fear that you might be HIV positive" (C4900, Male aged 20-30, Group D).

Another participant who did not test described feeling overwhelmed by the reliance on medication that would be implied by a potentially positive result.

Interviewer: *'What are your concerns about HIV?'*
Participant: *'I think it's the fact that once you become HIV positive you must know that you will be taking medication for the rest of your life. Yes, you can live long but knowing that you will be taking medication for the rest of your life is what concerns me'.* (C3061, Female aged 20-30, Group E).

Based on these barriers, the stakeholder group agreed that the intervention needed to increase confidence that treatment is effective and accessible, and that being HIV positive does not have to mean sickness or losing your status and respect within the community. The group proposed that this education could be delivered by HIV positive peer mentors discussing how treatment helps keep them healthy, as having a role model was theorised to be more effective than information only.

3.1.2 Couples counselling sessions need to give couples a safe space to discuss perceived barriers to CHTC

The interviews with couples counsellors suggested that their investment in positive outcomes for the couple could lead them to provide advice and try to persuade couples to engage in CHTC.'*you speak and speak and speak and encouraging them about honesty and trust and everything but still tomorrow, no, I'm not ready to do the testing..... I kept on encouraging them that most of the people are testing negative, just to make them think about it*' (Staff member 1, counsellor and facilitator)

For peer review only - http://bmjopen.bmj.com/site/about/guidelines.xhtml

Some participants also seemed to expect that the counselling session would involve advice:

'*To get a chance with a third person who is going to advise you on how things should happen'*. (C680, Male aged 30-40, Group B)

The stakeholder group proposed that training facilitators in the rationale for using open questioning could help encourage them to use this technique more often, and thereby enable a couple to discuss why they are not ready to test together and to come up with their own solutions. Open questioning involves asking the couple open-ended questions, such as "What makes it harder for you to test together?", to enable the couple to discuss their barriers. Training in this technique would encourage facilitators that it is not their responsibility to ensure couples engage in CHTC, and that their success as facilitators is not evaluated based on CHTC uptake. The Healthy Conversations Skills Training package (23) was identified as an appropriate tool to deliver this message during facilitator training. This emphasises to facilitators that their role is to help people recognise their own barriers and to ask open discovery questions to facilitate them in identifying solutions.

3.1.3 Couples need to feel ready to have conversations about CHTC outside the intervention setting

Couples in the Uthando Lwethu intervention were encouraged to discuss HIV testing with one another outside the intervention setting. However, a common barrier was that discussing HIV testing outside the supportive environment of the intervention was difficult due to the implied lack of trust.

'*Yes, we would talk about it [testing]. He said that it means that I do not trust him, why else would I talk about this thing. You would see from the way he responds that you should not go any further with it.'* (C0011, female aged 20-30 years, Group D)

This contrasts with a quote from a participant who tested with his partner after only one counselling session, describing their rationale for testing together:

For peer review only - http://bmjopen.bmj.com/site/about/guidelines.xhtml

‘*If she is going to come back already tested while I have not tested, there might be finger-pointing, but if we find out at the same time, we might get the same shock and be able to comfort each other, and then look for a way forward*’. (C4510, male aged 40-50 years, Group A)

Concerns about trust and blame were reflected in the staff interviews too.

‘*I think there’s a lot of barriers when in a couple there’s STI because there’s a lot of blame. There’s a lot of feeling that someone is having an affair so people they don’t…I think they won’t be comfortable*’ (Staff member 4, counsellor and facilitator)

The stakeholder group identified several optimisations to the intervention which could facilitate couples to feel ready to discuss CHTC without blame. These included education about serodiscordance to overcome perceptions that infection automatically indicates infidelity, and a case study of a serodiscordant couple was identified as an effective means to convey this message. In addition, it was agreed that inviting peer mentors from the local community to single-gender sessions could help provide information from a credible, relatable source and facilitate a dialogue about barriers to couples testing.

As well as optimising the content of the intervention, the group also proposed optimising the structure to facilitate couples to feel ready to test together. The original intervention involved one single-gender group session, which was highly valued by men and women as a chance to talk openly about relationship issues.

‘*Do you know how difficult it is to talk when your boyfriend is here? You cannot be open to talk about whatever, but when you are with other women you can talk about whatever concerning him*’ (C0011, Female aged 20-30 years, Group D)

Staff members also noticed that participants were more willing to have open discussions during the single-gender group session. Therefore, the stakeholder group agreed that the total time for participants in single-gender sessions could be increased in order to facilitate an open dialogue

For peer review only - http://bmjopen.bmj.com/site/about/guidelines.xhtml

around barriers to HIV testing and associated implications at an earlier point in the intervention. This was theorised to help couples feel more ready to discuss testing together outside the intervention.

3.2 What are the theorised mechanisms of action through which the optimised intervention can change behaviour?

In order to inform the process of optimising the intervention, guiding principles were developed based on these findings regarding the main barriers to CHTC, see Table 3. These emphasise that the intervention needs to increase intimacy between couples, encourage effective communication about CHTC, and increase understanding.

<Insert Table 3 here>

A logic model was developed to show how this optimised intervention is theorised to promote CHTC uptake (Figure 2). As in Uthando Lwethu, transforming motivations remains a key mechanism through which the intervention seeks to increase CHTC uptake. This construct comes from communal coping theory (24), which proposes that by improving relationship functioning and communication within a couple, motivation for looking after health can be shifted from the individual to the couple. In Uthando Lwethu, this approach informed the couples counselling sessions which aimed to improve relationship functioning and communication skills, thereby facilitating difficult discussions that partners may wish to have and indirectly influencing CHTC uptake.

The optimised intervention still seeks to promote transformation of motivation amongst couples, but alongside this it has a more direct focus on increasing understanding of the consequences of testing, changing outcome expectancies, and managing negative emotions around HIV testing. This was informed by the qualitative interviews which suggested some people needed further support to feel ready to test for HIV. Therefore, the optimised intervention is theorised to operate both through

For peer review only - http://bmjopen.bmj.com/site/about/guidelines.xhtml

an indirect pathway of improving relationship functioning and intimacy, but also through a more direct pathway of overcoming barriers to CHTC. Key differences from Uthando Lwethu are shown in red.

<Insert Figure 2 here>

4. Discussion

This study adopted the person-based approach to explore couples' and facilitators' experiences and perceptions of an intervention to promote CHTC uptake in KZN, South Africa.

Suggested optimisations identified with a stakeholder and community group to promote CHTC included group discussion facilitated by peer mentors to help improve outcome expectancies about living with HIV and address concerns about the impact on relationships, a more explicit focus on discussing barriers to CHTC during couples' counselling sessions, and more time in single-gender sessions in order to facilitate open discussion about HIV.

The use of rigorous qualitative methods was a strength of this study as it provided in-depth understanding of facilitators and barriers to couples' HIV testing, which informed optimisation of the intervention components. This is in line with recommendations that qualitative research be used to explore how HIV interventions might change behaviour to help enhance effectiveness (25). A significant limitation was that the interviews were conducted approximately five years after the intervention was delivered. Whilst qualitative interviews always rely on the reconstruction of experiences through the participant's narrative lens (26), the significant time gap meant that the experiences shared with us were particularly subject to the influence of subsequent events and the passing of years, and should be interpreted carefully. The discussion with the community group helped confirm that the findings are relevant. In an ongoing trial of the optimised intervention,

For peer review only - http://bmjopen.bmj.com/site/about/guidelines.xhtml

For peer review only

process interviews will be conducted to assist in understanding barriers and facilitators to CHTC at the time couples are making this decision.

While participants openly discussed perceived barriers to CHTC, very few responded to questions about aspects of the intervention they disliked. The use of vignettes about a fictional person may have helped participants feel comfortable to provide more open feedback.

Our finding that couples can find it difficult to discuss CHTC without connotations of blame is consistent with qualitative research in Sub-Saharan Africa showing that people overestimate the likelihood of becoming infected after a one-off sexual encounter and are therefore reluctant to test due to concerns that the test will reveal they have been unfaithful (14).The perception that discordancy is related to infidelity has been theorised to be exacerbated by social norms for multiple partners; low marriage rates; campaigns that associate HIV prevention with monogamy; and lack of knowledge about the potential for long-term differences in HIV status within couples represented by serodiscordance (13).

Misunderstandings about serodiscordance and the associated blame presents a significant challenge to implementing CHTC, and supports the need for a supportive environment for couples to discuss HIV testing, with a focus on ensuring the best future together rather than considering past infidelities, as well as education about serodiscordance. This provided a strong rationale for the theoretical shift in the logic model towards addressing perceived barriers more directly, by increasing knowledge of HIV infection and possible outcomes of testing, and exploring negative emotions around CHTC. This is in line with Protection Motivation Theory (27), in that the optimised intervention seeks to influence couples' threat perceptions and increase their self-efficacy for coping with the threat. However, unlike many interventions which aim to raise perceived risk to change behaviour, this intervention seeks to reduce the perceived risk that CHTC will threaten a couple's trust. As well as being grounded in theory, the optimisations proposed by this study are evidence-

For peer review only

based as effective strategies for facilitating CHTC uptake, including understanding the benefits of CHTC, and inviting peer mentors to facilitate discussions (28, 29).

The study offered novel insights into the experiences of lay counsellors from the community delivering a couples-focused intervention for HIV testing. As asking open questions is known to be effective to support behaviour change, whilst telling or giving advice can lead to disagreement from the recipient (30), the findings from this study suggested that additional training for counsellors to support open questioning and absolve personal responsibility for couples' decisions may be beneficial.

The optimised intervention will be evaluated in a study in KZN, and CHTC uptake will be compared with uptake in the intervention arm of the original RCT. The process of optimisation could be further streamlined in future research by adopting a flexible, adaptive trial design that allows modifications to be made in line with ongoing qualitative process research, such that barriers can be addressed as they come to light (31).

5. Conclusions

In-depth qualitative interviews identified key barriers to engaging with CHTC, including concerns about trust in the relationship, and a lack of open discussion about barriers to CHTC during counselling sessions. This informed proposed optimisations to an existing couples-focused intervention in line with evidence and theory to overcome these barriers, including the addition of a discussion with peer mentors, education about serodiscordance, and training facilitators to use open questions to explore couples' barriers to CHTC. The use of the person-based approach to intervention optimisation ensured that these changes were grounded in participants' experiences and psychosocial context.

Acknowledgements:

For peer review only

Thank you to all participants in this research study.

The following people have provided helpful comments or suggestions, or have otherwise enabled this work: Dr Tawanda Makusha (HSRC), and Prof Lynae Darbes (University of Michigan).

Funding:

This report is independent research supported by the National Institute for Health Research using Official Development Assistance (ODA) funding (NIHR Global Health Research Professorship, Professor Nuala McGrath, RP-2017-08-ST2-008). KM, TM, LM, NN, ZF and NMcG were supported by this funding. The views expressed in this publication are those of the author(s) and not necessarily those of the NHS, the National Institute for Health Research or the Department of Health and Social Care.

Competing interests: The authors have no competing interests to declare.

Authors' contributions: LM and TM conducted the interviews. KM trained the interviewers, conducted some staff interviews, analysed the data, and wrote the manuscript. AVH, TN, PJ, VH, HVR and NM contributed to the original intervention design and conducted the 'Uthando Lwethu' trial, as well as planning and organising the present qualitative study. NN coordinated the participant recruitment, data collection and community working group and participant feedback events. ZF contributed to intervention optimisation. All authors contributed to decisions about intervention optimisations and reviewed the manuscript.

Data sharing statement: The interview transcripts will not be made available to protect participants' anonymity.

For peer review only - http://bmjopen.bmj.com/site/about/guidelines.xhtml

References

1. World Health Organization. Guidance on couples HIV testing and counselling including antiretroviral therapy for treatment and prevention in serodiscordant couples: recommendations for a public health approach. 2012.
2. National Department of Health. National HIV Testing Services: Policy. 2016.
3. Kilembe W, Wall KM, Mokgoro M, Mwaanga A, Dissen E, Kamusoko M, et al. Implementation of couples' voluntary HIV counseling and testing services in Durban, South Africa. BMC Public Health. 2015;15(1):601.
4. Wall K, Allen S. Incentives to improve couples' HIV testing uptake and cost-effectiveness. The Lancet Global Health. 2017;5(9):e847-e8.
5. Vandormael A, Cuadros D, Kim HY, Barnighausen T, Tanser F. The state of the HIV epidemic in rural KwaZulu-Natal, South Africa: a novel application of disease metrics to assess trajectories and highlight areas for intervention. Int J Epidemiol. 2020.
6. Kim HY, Harling G, Vandormael A, Tomita A, Cuadros DF, Barnighausen T, et al. HIV seroconcordance among heterosexual couples in rural KwaZulu-Natal, South Africa: a population-based analysis. J Int AIDS Soc. 2020;23(1):e25432.
7. Matthews LT, Moore L, Crankshaw TL, Milford C, Mosery FN, Greener R, et al. South Africans with recent pregnancy rarely know partner's HIV serostatus: implications for serodiscordant couples interventions. BMC Public Health. 2014;14(1):843.
8. World Health Organization. Guideline on when to start antiretroviral therapy and on pre-exposure prophylaxis for HIV. 2015.
9. United Nations General Assembly. United Nations General Assembly. Political Declaration on HIV and AIDS: on the Fast-Track to accelerating the fight against HIV and to ending the AIDS epidemic by 2030. . New York, USA2016.
10. Darbes LA, McGrath NM, Hosegood V, Johnson MO, Fritz K, Ngubane T, et al. Results of a couples-based randomized controlled trial aimed to increase testing for HIV. JAIDS Journal of Acquired Immune Deficiency Syndromes. 2019;80(4):404-13.
11. Yardley L, Ainsworth B, Arden-Close E, Muller I. The person-based approach to enhancing the acceptability and feasibility of interventions. Pilot and feasibility studies. 2015;1(1):37.
12. Yardley L, Morrison L, Bradbury K, Muller I. The person-based approach to intervention development: application to digital health-related behavior change interventions. J Med Internet Res. 2015;17(1):e30.
13. Parker L, Pettifor A, Maman S, Sibeko J, MacPhail C. Concerns about partner infidelity are a barrier to adoption of HIV-prevention strategies among young South African couples. Cult Health Sex. 2014;16(7):792-805.
14. Conroy AA. 'It means there is doubt in the house': perceptions and experiences of HIV testing in rural Malawi. Culture, Health & Sexuality. 2014;16(4):397-411.
15. Pettifor A, MacPhail C, Nguyen N, Rosenberg M, Parker L, Sibeko J. Feasibility and acceptability of Project Connect: a couples-based HIV-risk reduction intervention among young couples in Johannesburg, South Africa. AIDS Care. 2014;26(4):476-82.
16. Duncan E, O'Cathain A, Rousseau N, Croot L, Sworn K, Turner KM, et al. Guidance for reporting intervention development studies in health research (GUIDED): an evidence-based consensus study. BMJ Open. 2020;10(4):e033516.
17. Darbes LA, van Rooyen H, Hosegood V, Ngubane T, Johnson MO, Fritz K, et al. Uthando Lwethu ('our love'): a protocol for a couples-based intervention to increase testing for HIV: a randomized controlled trial in rural KwaZulu-Natal, South Africa. Trials. 2014;15(1):64.
18. Markman HJ, Renick MJ, Floyd FJ, Stanley SM, Clements M. Preventing marital distress through communication and conflict management training: a 4- and 5-year follow-up. J Consult Clin Psychol. 1993;61(1):70-7.

For peer review only - http://bmjopen.bmj.com/site/about/guidelines.xhtml

19. Bradbury K, Morton K, Grist R, Band R, Van Woezik A, Connelly D, et al. Using the Person-Based Approach to optimise a digital intervention for the management of hypertension. PLoS One. 2018;13(5):e0196868.
20. Bradbury K, Watts S, Arden-Close E, Yardley L, Lewith G. Developing digital interventions: a methodological guide. Evid Based Complement Alternat Med. 2014;2014.
21. Atkins L, Francis J, Islam R, O'Connor D, Patey A, Ivers N, et al. A guide to using the Theoretical Domains Framework of behaviour change to investigate implementation problems. Implementation Science. 2017;12(1):77.
22. Michie S, Van Stralen MM, West R. The behaviour change wheel: A new method for characterising and designing behaviour change interventions. Implementation Science. 2011;23(6):42.
23. Vogel C, Lawrence W, Cradock S, Ntani G, Tinati T, Jarman M, et al. Healthy Conversation Skills: increasing competence and confidence in front-line staff. Public Health Nutr. 2012;17:1-8.
24. Lewis MA, McBride CM, Pollak KI, Puleo E, Butterfield RM, Emmons KM. Understanding health behavior change among couples: an interdependence and communal coping approach. Soc Sci Med. 2006;62(6):1369-80.
25. Camlin CS, Seeley J. Qualitative research on community experiences in large HIV research trials: what have we learned? J Int AIDS Soc. 2018;21 Suppl 7:e25173.
26. Randall W, Phoenix C. The problem with truth in qualitative interviews: reflections from a narrative perspective. Qualitative Research in Sport and Exercise. 2009;1:125-40.
27. Rogers RW. Cognitive and psychological processes in fear appeals and attitude change: A revised theory of protection motivation. Social psychophysiology: A sourcebook. 1983:153-76.
28. Nannozi V, Wobudeya E, Matsiko N, Gahagan J. Motivators of couple HIV counseling and testing (CHCT) uptake in a rural setting in Uganda. BMC Public Health. 2017;17(1):104.
29. Adeomi AA, Adeoye OA, Asekun-Olarinmoye EO, Abodunrin OL, Olugbenga-Bello AI, Sabageh AO. Evaluation of the Effectiveness of Peer Education in Improving HIV Knowledge, Attitude, and Sexual Behaviours among In-School Adolescents in Osun State, Nigeria. AIDS Res Treat. 2014;2014:131756.
30. Mason P, Butler C. Health Behavior Change: A Guide For Practitioners2010.
31. Pallmann P, Bedding AW, Choodari-Oskooei B, Dimairo M, Flight L, Hampson LV, et al. Adaptive designs in clinical trials: why use them, and how to run and report them. BMC Med. 2018;16(1):29-.

For peer review only

For peer review only - http://bmjopen.bmj.com/site/about/guidelines.xhtml

Table 1. Uthando Lwethu intervention content.

Session	Content
Group session 1	A mixed gender half-day session with approximately 15-20 couples. Information provision on TB, HIV, contraception and alcohol. This was designed as a baseline session for all couples before randomisation, and was not not part of the intervention content. Couples were randomised to the intervention or control group at the end of this session.
Group session 2	A single gender half-day session including discussions about relationship dynamics, HIV treatment, gender norms, and practical skills sessions on using condoms and learning communication techniques.
Couples counselling session 1	A 90-120 minute counselling session including discussion of relationship expectations, communication skills and goal-setting.
Couples counselling session 2	A 90-120 minute counselling session to develop communication skills, discuss barriers to achieving their goals and engage in problem-solving, and enhance positive relationship dynamics and intimacy using activities to help focus on what they like about their relationship and their partner.
Couples counselling session 3	A 90-120 minute counselling session to continue building communication and problem-solving skills.
Couples counselling session 4	A 90-120 minute counselling session to discuss maintenance of any behaviour changes, goal-setting for the future and identify possible challenges they might encounter that could impact their relationship.

For peer review only

For peer review only - http://bmjopen.bmj.com/site/about/guidelines.xhtml

Table 2. Excerpt from Table of Changes analysis.

Intervention Component	Participant	Positive comments	Negative comments	Possible Change	Reason for Change IMP; EAS; REP; EXP; NCON; NC	MoSCoW MUST do SHOULD do COULD do WOULD like
Single gender session	4511, Female aged 30-40, group A	It would be girls alone and boys by themselves and you would talk about the problems that you come across with your partner. And you would get solutions for if the problem occurs again, this is how you could face it. Interviewer: Oh. So, how was it? Participant: It was fun because we were comfortable, you could talk comfortably. There was no one with a secret.		Increase the amount of time spent in single gender sessions to allow more opportunity for open discussion amongst peers and help couples feel more ready for testing.	IMP, REP	Should have
CHTC	3960, Male aged 30-40, group D		I think if you check simultaneously with your partner, if there come different results, its better different results come out if you are not testing together and you will have to answer why results are like that and how to solve such a problem if it arises	Fear of serodiscordance needs to be addressed, perhaps through peer mentors, education about serodiscordance.	IMP	Must

For peer review only - http://bmjopen.bmj.com/site/about/guidelines.xhtml

Table 3. Guiding principles to inform the optimisation of the intervention

Design objectives	Key intervention features
Help couples feel close to each other, recognising the positive aspects of their relationship and providing motivation to look after their health together.	- Activities to promote focus on the positive aspects of the relationship.
Encourage effective communication to address fears and outcome expectancies about testing.<br><br>Address negative emotions.	- Ask open questions to enable couples to identify their barriers to CHTC and come up with their own solutions.<br>- Provide effective communication skills training to help couples explore their perceived barriers to testing together.
Increase understanding about risk of HIV transmission, serodiscordance, and effectiveness of treatment.	- Interactive single-gender group activities<br>- Open dialogue facilitated by a peer mentor with experience of couples HIV testing<br>- Provide standard guidance on HIV prevention to reduce risk of transmission, e.g. condom demonstration.

For peer review only - http://bmjopen.bmj.com/site/about/guidelines.xhtml

Figure legends:

Figure 1. Stages of research leading up to and from present optimisation study.

Figure 2. Logic model.

For peer review only

For peer review only - http://bmjopen.bmj.com/site/about/guidelines.xhtml

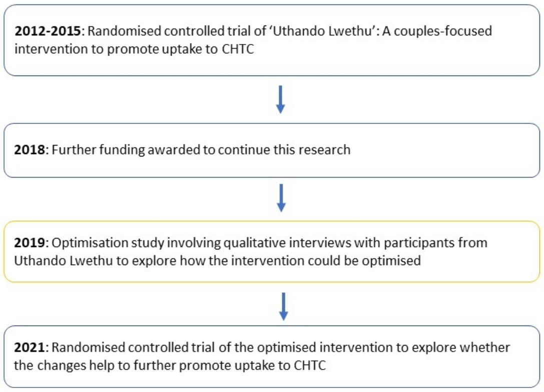

Figure 1. Stages of research leading up to and from present optimisation study.

338x190mm (96 x 96 DPI)

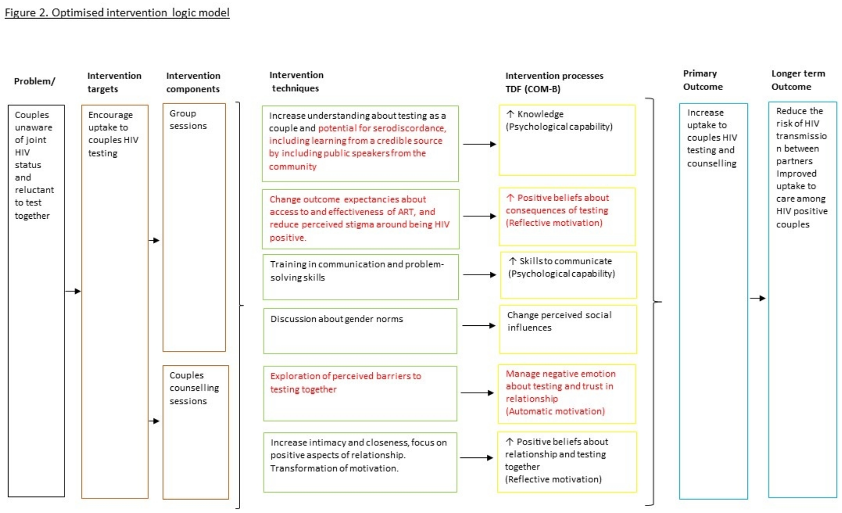

Figure 2. Optimised intervention logic model.

338x190mm (96 x 96 DPI)

Consolidated criteria for reporting qualitative studies (COREQ): 32-item checklist

Developed from:
Tong A, Sainsbury P, Craig J. Consolidated criteria for reporting qualitative research (COREQ): a 32-item checklist for interviews and focus groups. *International Journal for Quality in Health Care*. 2007. Volume 19, Number 6: pp. 349 – 357

YOU MUST PROVIDE A RESPONSE FOR ALL ITEMS. ENTER N/A IF NOT APPLICABLE

No. Item	Guide questions/description	Reported on Page #
**Domain 1: Research team and reflexivity**		
Personal Characteristics		
1. Interviewer/facilitator	Which author/s conducted the interview or focus group?	Page 9 and 10
2. Credentials	What were the researcher's credentials? E.g. PhD, MD	Page 9 and 10
3. Occupation	What was their occupation at the time of the study?	Page 9 and 10
4. Gender	Was the researcher male or female?	Page 9 and 10
5. Experience and training	What experience or training did the researcher have?	Page 9
Relationship with participants		
6. Relationship established	Was a relationship established prior to study commencement?	8
7. Participant knowledge of the interviewer	What did the participants know about the researcher? e.g. personal goals, reasons for doing the research	Supplementary Files 2 and 3
8. Interviewer characteristics	What characteristics were reported about the interviewer/facilitator? e.g. Bias, assumptions, reasons and interests in the research topic	Supplementary Files 2 and 3
**Domain 2: study design**		
Theoretical framework		
9. Methodological orientation and Theory	What methodological orientation was stated to underpin the study? e.g. grounded theory, discourse analysis, ethnography, phenomenology, content analysis	Page 7 and 10
Participant selection		
10. Sampling	How were participants selected? e.g. purposive, convenience, consecutive, snowball	Page 8
11. Method of approach	How were participants approached? e.g. face-to-face, telephone, mail, email	Page 9

For peer review only

12. Sample size	How many participants were in the study?	Page 11
13. Non-participation	How many people refused to participate or dropped out? Reasons?	Page 9
Setting		
14. Setting of data collection	Where was the data collected? e.g. home, clinic, workplace	Page 9
15. Presence of non-participants	Was anyone else present besides the participants and researchers?	N/A
16. Description of sample	What are the important characteristics of the sample? e.g. demographic data, date	Page 11
Data collection		
17. Interview guide	Were questions, prompts, guides provided by the authors? Was it pilot tested?	Supplementary Files 2 and 3
18. Repeat interviews	Were repeat inter views carried out? If yes, how many?	N/A
19. Audio/visual recording	Did the research use audio or visual recording to collect the data?	Page 9
20. Field notes	Were field notes made during and/or after the inter view or focus group?	Page 9
21. Duration	What was the duration of the inter views or focus group?	Page 11
22. Data saturation	Was data saturation discussed?	N/A
23. Transcripts returned	Were transcripts returned to participants for comment and/or correction?	N/A
**Domain 3: analysis and findings**		
Data analysis		
24. Number of data coders	How many data coders coded the data?	Page 10
25. Description of the coding tree	Did authors provide a description of the coding tree?	N/A
26. Derivation of themes	Were themes identified in advance or derived from the data?	N/A
27. Software	What software, if applicable, was used to manage the data?	N/A
28. Participant checking	Did participants provide feedback on the findings?	Page 10
Reporting		
29. Quotations presented	Were participant quotations presented to illustrate the themes/findings? Was each quotation identified? e.g. participant number	Page 12-14
30. Data and findings consistent	Was there consistency between the data presented and the findings?	Yes
31. Clarity of major themes	Were major themes clearly presented in the findings?	Yes
32. Clarity of minor themes	Is there a description of diverse cases or discussion of minor themes?	Yes

For peer review only

Once you have completed this checklist, please save a copy and upload it as part of your submission. When requested to do so as part of the upload process, please select the file type: *Checklist*. You will NOT be able to proceed with submission unless the checklist has been uploaded. Please DO NOT include this checklist as part of the main manuscript document. It must be uploaded as a separate file.

For peer review only

Supplementary File 2: Semi-structured interview schedule for participants in the couples-focused intervention

**A. Introduction and thanks**
My name is ************ and I am working for the 'Egugu Lethu project' which is being conducted by the Human Sciences Research Council and the University of Southampton, UK.<br><br>First, let me thank you for agreeing to speak to me today.
**B. Project explanations**
The overall aim of the 'Egugu Lethu project is to improve the couples counselling intervention that we used in the Uthando Lwethu study and to conduct a new study to test the improved intervention success in promoting couples HIV testing and counselling.<br><br>This interview is part of the first steps towards improving the previously tested intervention. We are conducting interviews with individual members of couples from the Uthando Lwethu study to explore their experiences of the intervention and it's role, if any, in contributing to their decision regarding uptake of couples HIV testing and counselling.

Interview explanations

Today I would like to ask you about your experiences of participating in the Uthando Lwethu Study. We would really like to hear your experiences, opinions and observations. I will ask you a series of questions from my guideline, but please feel free to add in extra comments that you think are important.
[I will be taping / I will not be taping] this interview and taking a few notes to aid my memory as we go along but I will not write down your name or the name of your partner.
I will not be using a questionnaire but have a list of topics that I'd like us to try and cover today. Think of our session as a conversation and that you can ask me questions too.

Great, so now we have completed the formalities, is it fine with you that we start?

What is your name?

And the name of your partner who participated with you in Uthando Lwethu Study?

General experience of the Uthando Lwethu Study

Before we start talking in detail about your experiences of specific parts of the Uthando Lwethu Study, can you tell me about the first day when you were approached about the study? What was that like for you?
Can you remember why you decided to sign up for the study?

For peer review only - http://bmjopen.bmj.com/site/about/guidelines.xhtml

I'm really interested in hearing about your experiences of the Uthando Lwethu study, Can you tell me what you remember about being in the study?

How did you find the sessions?

Does anything stand out in your memory about the study? What do you remember and why?

Can you tell me about what difference Uthando Lwethu made for you, if any?

Experience of the first group session

As a quick reminder, the first group session was where we gave some information about health to the group of couples attending together and at the end we had the lottery where you learned you and your partner were going to receive the Uthando Lwethu couples counselling sessions .

Can you tell me all about what the first group session was like for you?

[Note to interviewer: information given was on TB, reproductive health/HIV), and a list of community resources for health care and treatment was given]

What information did you find most helpful? And why?

What other information would you have liked?

How was it to see other couples from your area at the same session? Did you know anyone else there? Who?

Can you remember how you sat in the first session? Did you sit with your partner, or with other men/women?

How did you interact with other couples from your area?

Can you tell us about anything you remember learning in the first session?

Experience of the second group session

The second group session was where you met in [men-only / female only] groups and discussed roles, violence and power within relationships. There was also skills training for communication and risk reduction. What was your experience of that second group session?

What was most helpful in this session? Why?

What other information would you have liked? Why?

For peer review only - http://bmjopen.bmj.com/site/about/guidelines.xhtml

Can you tell me about any conversations you had with your partner about that session afterwards?

What did you discuss? Once off or repeatedly?

Were there any particular topics that you discussed in particular? [prompt for specific exercises / content that was included in the session to help participants remember]

Was one same gender group session enough or would you have liked more? Why?

Couples sessions

The next section is dependent on whether they attended the couples' sessions. Only one block of questions from this section is applicable in any particular individual interview.

a. For those who did NOT attend couples sessions at all

If you remember, you were then offered 4 couples' sessions *with* <counsellor's name> Can you tell me about why you did not attend the couples' sessions that were on offer?

How did you reach that decision?

Was this your choice or your partner's, or mutual?

What were your main concerns about the couples' sessions?

Could the study have done something differently that might have changed your mind? Or your partner's mind? Or made it possible logistically?

b. For those who attended one or more couples sessions

What was your main reason for deciding to attend the couples' sessions?

How did you reach that decision?

Did you have any concerns or worries about attending?

Do you remember your counsellor? [check they mean the same person recorded on page 1]. I am interested in how you felt about the couples' sessions that you attended.

If I asked you to describe ~~them~~ *the sessions in one or two sentences*, what would you say?

For peer review only - http://bmjopen.bmj.com/site/about/guidelines.xhtml

*Did you feel you were able to communicate your point of view and thoughts to your **partner** in the counselling session? And did your partner? Can you tell me about why?*

*Did you feel you were able to communicate your point of view and thoughts to the **counselor** in the counselling session? Can you tell me about why?*

How do you think your partner felt about it?

Was there anything you would change?

***[If they used all 4 sessions]:** Was the number of sessions enough*

***[If they used less than 4 sessions]:** Can you tell me about why you stopped attending when you did?*

Would having an additional session made a difference? Was another session likely to have changed your decision about testing?

Can you tell us about anything you remember learning from the sessions? [prompt for specific tasks or exercises / content to help participants remember]

Did you discuss anything about the session or from a session with your partner afterwards? What did you talk about?

Do you remember some of the homework exercises that the counsellor gave you? How did you and your partner get on with the homework?

(If still together) Can you tell me how you feel about communicating with your partner now?

Are you still practicing the skills taught? Why or why not? If not, how long after did you stop using the skills?

Cohabitation

The next section is dependent on the couple cohabitation status at the time of their participation in the Uthando Lwethu study. <u>Only one block</u> of questions from this section is applicable in any particular individual interview.

a. For those cohabiting at the time

At the time of the Uthando Lwethu study, you were living with your partner.

Can you tell me about what it was like doing the Uthando Lwethu study while you were living at home with your partner?

Did you want to talk to your partner about Uthando Lwethu?

If yes – did you have the opportunity to talk to them at home?

If no – why not?

Was anything different at home when you were both doing Uthando Lwethu?

For peer review only - http://bmjopen.bmj.com/site/about/guidelines.xhtml

b. For those NOT cohabiting at the time

At the time of the Uthando Lwethu study, you were not living with your partner.

Can you tell me about what it was like doing the Uthando Lwethu study while you were living separately from your partner?

Did you want to talk to your partner about Uthando Lwethu?

If yes – did you have the opportunity to talk to them? Where did the conversations happen?

If no – why not?

Was anything different when you met up while you were both doing Uthando Lwethu?

HIV testing

The next section is for ALL individuals

What do you think about HIV testing?

And testing with a partner together?

What are your main concerns about HIV testing?

How could we address those concerns?

The next section is dependent on whether the couple tested for HIV as a couple during the Uthando Lwethu study. Only one block of questions from this section is applicable in any particular individual interview.

a. For those who did not test for HIV as a couple during the Uthando Lwethu Study

I'm really interested in your decision around whether to test for HIV with your partner. It looks like you decided not to test together during the study, can you tell me more about that decision?

Did you as a couple discuss testing? Why or why not? How did you reach a decision?
How did you feel about getting tested?

How did your partner feel?

For peer review only - http://bmjopen.bmj.com/site/about/guidelines.xhtml

What were your main concerns? What could be done to address those concerns? Eg more information? sessions? support?

What do you think might have been the benefits of testing for you?

How did you resolve any conflict with your partner?

Did you test together anytime after the study had finished? How long after the study?

Do you know your partner's status? How do you know this?

Do you think the group sessions or couples counseling sessions contributed to your / your partner's decision?

b. For those who tested for HIV as a couple during the Uthando Lwethu Study

Have you ever tested together before Uthando Lwethu?
If yes - how did you decide to do this?

Can you tell me about why you decided to test together during the Uthando Lwethu study?

How did you reach a decision?

How did you feel about getting tested?

How did your partner feel?
Did you have any concerns about the testing? What could be done to address those concerns? Eg more information? sessions? support?

How did you resolve any conflict with your partner about deciding whether to get tested?

How did you arrange the testing?

How did you feel after the testing?

Did you talk about it with your partner? What do you think were the benefits of testing for you?

How would you encourage other couples to have HIV testing?

For peer review only - http://bmjopen.bmj.com/site/about/guidelines.xhtml

Do you think the group sessions or couples counseling sessions contributed to your / your partner's decision?

If yes: What part of the intervention encouraged you to be tested?

If not, why not?

For all individuals

In the new study, we may offer testing for sexually transmitted infections (STIs), specifically testing for chlamydia, syphilis, gonorrhoea and trichomoniasis. However we don't know if this would be useful for people, or what some of the concerns might be in having STI testing.

Can you tell me what you think about the idea of testing couples for sexually transmitted infections?

If we had also invited you to consider STI testing as part of the couples HIV testing session, how would you have felt about that?

Would you have taken up an offer for STI testing? Why/why not?

The STI testing would mean giving a sample that would be sent away to the labs for testing and you and your partner would come back to receive the results, For men, this would involve providing a urine sample. How would you feel about that?

For women, this would mean privately taking a vaginal swab. Many women do this themselves, or the nurse could help you with if you wanted. How would you feel about that?

Overall, what do you think about the Uthando Lwethu study for other people?

We are hoping to make some changes to Uthando Lwethu to make it even better for people. Can you think of any other health or relationship topics you would have liked to know more about in group session 1, group session 2, or the counselling sessions?

Can you think of anything that would have made Uthando Lwethu better for you?

And was there any part of Uthando Lwethu that you think was not needed or was not helpful to you?

For peer review only - http://bmjopen.bmj.com/site/about/guidelines.xhtml

Supplementary File 3. Semi-structured interview schedule for staff members.

**A. Introduction and thanks**
My name is ************ and I am working for the 'Egugu Lethu project' which is being conducted by the Human Sciences Research Council and the University of Southampton, UK.<br><br>First, let me thank you for agreeing to speak to me today.
**B. Project explanations**
The overall aim of the Egugu Lethu project is to improve the couples counselling intervention that we used in the Uthando Lwethu study and to conduct a new study to test the improved intervention success in promoting couples HIV testing and counselling.<br><br>The first step of optimising the previously tested intervention is to conduct interviews with individual members of couples from the Uthando Lwethu study and with staff members who were involved in delivering the intervention components. In staff interviews we will discuss staff experiences of the previous study and possible barriers to couples' decisions to test for HIV together.

Interview explanations

Today I would like to ask you about your experiences as an Uthando Lwethu Study staff member. We would really like to hear your experiences, opinions and observations. I will ask you a series of questions from my guideline, but please feel free to add in extra comments that you think are important. [I will be taping / I will not be taping] this interview and taking a few notes to aid my memory as we go along but I will not write down your name. I will not be using a questionnaire but have a list of topics that I'd like us to try and cover today. Think of our session as a conversation and that you can ask me questions too.

Great, so now we have completed the formalities, is it fine with you that we start?

What is your name?

What was your role in the Uthando Lwethu Study: Couples counsellor or part of the group sessions or counsellor for the HIV testing and counselling?

Can you tell me a bit about your background? I'd like to understand what your job is outside of Uthando Lwethu?
Can you tell me about the training you've had previously?
What was different in Uthando Lwethu from your usual counselling/facilitating role?
What was the same?

General experience of the Uthando Lwethu Study

For peer review only

Before we start talking in detail about your experiences and perceptions of specific parts of the Uthando Lwethu Study, I would like to hear what was it like being part of the study team overall?

How did you find the training you received before becoming a facilitator/counsellor for Uthando Lwethu?

The next section is dependent on which role the staff member had. <u>Only one block</u> of questions from this section is applicable in any particular individual interview.

For staff who attended group sessions

How many first group sessions did you attend? What was your role in the first group sessions?

what was your role in the second group sessions?

We're interested in how to improve the intervention for couples, so the next few questions are to try and find out more about how it worked last time, and what we could do better going forwards.

How did you find the first group sessions?

How did you find the second group sessions?

Did you notice any differences between the two sessions?

Can you recall what happened in a typical second group session?

What was it like following the manual for Uthando Lwethu during the second group session?

Were there any activities that stood out for you as being particularly important in the second group session?

Were there any activities that you remember being challenging for some people in the second group session?

How engaged did you feel people were? Can you tell me a story about any memorable moments you recall?

Are there any particular benefits/negatives to the single gender session?

For peer review only - http://bmjopen.bmj.com/site/about/guidelines.xhtml

How did the single gender sessions vary between groups?

What was it like to deliver this information to females/males as a man/woman yourself? Did the same / different gender dynamic work well or not really?

Could session 2 be reinforced by homework If we introduced homework at this session? do you think it would work best done as a couple of individually?

Did you feel there were any differences in the level of participation or questions between sessions?

How important do you think these group sessions were for encouraging couples to attend HIV testing together?

Do you think there was anything about the first or second group session that didn't work very well, that you think we should change for the next study? *[prompt for organizational or staff changes vs content]*

If **not** already mentioned, ask about:

In the first group session, how do you think couples found the information on:

- TB
- HIV
- HIV testing
- Contraception
- Alcohol

In the second group session, how do you think participants found the:

- Discussion around long-term relationships
- Condom demonstration
- Discussions about gender and power imbalance
- Practising communication skills

In the new study, we may offer testing for sexually transmitted infections (STIs), specifically testing for chlamydia, syphilis, gonorrhoea and trichomoniasis. Would any changes be needed in the way Group sessions 1 and 2 are conducted or their content if STI testing were offered alongside HIV testing and counselling?

How do you think couples would feel about testing for STIs together? For men, it would involve providing a urine sample, and for women it would mean taking a vaginal swab. The samples are sent off to a lab, so the couple would need to come back to receive their results. How do you think this would work for couples in Uthando Lwethu?

For staff who conducted couples counselling sessions

For peer review only - http://bmjopen.bmj.com/site/about/guidelines.xhtml

For peer review only

Approximately, how many couples did you counsel?

How did you feel about the couples' counselling sessions that you led?

How do you think they were experienced by the participating couples?

Within those counselling sessions, how did you find it interacting with the female partner, as a female/male counsellor? And what about working with the male partner? Why?

Did the interaction with different couples vary? In what way did it vary and why?

Can you tell me about any reasons you can think of why some couples got more out of the counselling than others?

How do you think couples found the number of counselling sessions? Do you think having an additional session would make a difference / likely to have changed couples' decisions about testing?

How do you think the couples felt about practicing the speaker-listener technique between sessions?

Which exercises in the counselling sessions were particularly helpful for couples?

Were there any activities in the counselling sessions that you remember being challenging for some couples?

What was it like following the intervention manual in the couples counselling session?

If **not** already mentioned, ask the following:
In the couples counselling sessions, what do you think it was like for couples:

- Identifying and talking about their expectations and barriers around HIV testing
- Practising the speaker listener technique with their partner
- Planning solutions to problems together
- Setting some personal goals
- Doing the 'forgetting something good' activity, where they noticed what helpful things their partner has done for them recently
- Doing the 'words of the heart' activity where couples chose words that describe their relationship

If the participant only talks about positive examples where couples liked these things, ask:

- *Were there any couples who struggled to do that?*

For peer review only - http://bmjopen.bmj.com/site/about/guidelines.xhtml

- *If so, what was that like for you?*

Why do you think some couples didn't go for HIV testing? What do you think were the barriers for them?

Can you tell me about a couple who did go for testing? Why do you think they decided to get tested?

In the new study, we may offer testing for sexually transmitted infections (STIs), specifically testing for chlamydia, syphilis, gonorrhoea and trichomoniasis. Would any changes be needed in the way the couples counselling sessions are conducted or their content if STI testing were offered alongside HIV testing and counselling?

How do you think couples would feel about testing for STIs together?
For men, it would involve providing a urine sample, and for women it would mean taking a vaginal swab. The samples are sent off to a lab, so the couple would need to come back to receive their results. How do you think this would work for couples in Uthando Lwethu?

For peer review only

For peer review only - http://bmjopen.bmj.com/site/about/guidelines.xhtml

For staff who conducted couples HIV testing and counselling during the Uthando Lwethu Study

Approximately how many couples HIV testing and counselling sessions did you conduct during Uthando Lwethu Study?

How did you feel about the couples' HIV testing and counselling sessions that you conducted?

Did the couples discuss with you how they came to the decision to test for HIV together? What reason(s) did they give?

In the new study, we may offer testing for sexually transmitted infections (STIs), specifically testing for chlamydia, syphilis, gonorrhoea and trichomoniasisdo you think offering STI testing as part of the session would change couples' interest in testing for HIV together?

To what extent do you think including STI testing in the couples testing session would change this session for you as counsellor?

How do you think couples would feel about testing for STIs together? For men, it would involve providing a urine sample, and for women it would mean taking a vaginal swab. The samples are sent off to a lab, so the couple would need to come back to receive their results. How do you think this would work for couples in Uthando Lwethu?

For all staff at the end:

Did you have any contact with couples after ***their*** *participation in the study had ended?*

What was the nature of those interactions? [Probe for friendly / casual, neighbour, asking advice, sought out staff member's skill as counsellor etc.]

For peer review only - http://bmjopen.bmj.com/site/about/guidelines.xhtml
